# A pilot 1-year follow-up randomised controlled trial comparing metacognitive training to psychoeducation in schizophrenia: effects on insight

**DOI:** 10.1038/s41537-022-00316-x

**Published:** 2023-01-30

**Authors:** Javier-David Lopez-Morinigo, Adela Sánchez-Escribano Martínez, María Luisa Barrigón, Paula-Jhoana Escobedo-Aedo, Verónica González Ruiz-Ruano, Sergio Sánchez-Alonso, Laura Mata-Iturralde, Laura Muñoz-Lorenzo, Daniel Cuadras, Susana Ochoa, Enrique Baca-García, Anthony S. David

**Affiliations:** 1 Departamento de Psiquiatría, Universidad Autónoma de Madrid, Madrid, Spain; 2 Departamento de Psiquiatría, IIS-Fundación Jiménez Díaz, Madrid, Spain; 3 Centro de Investigación Biomédica en Red de Salud Mental, CIBERSAM, Madrid, Spain; 4 Department of Child and Adolescent Psychiatry, Institute of Psychiatry and Mental Health, Hospital General Universitario Gregorio Marañón, IiSGM, CIBERSAM, School of Medicine, Universidad Complutense, Madrid, Spain; 5 Department of Psychiatry, University Hospital Virgen del Rocio, Seville, Spain; 6 Etiopatogenia y tratamiento de los trastornos mentales graves (MERITT), Institut de Recerca Sant Joan de Déu, Barcelona, Spain; 7 Parc Sanitari Sant Joan de Déu, Barcelona, Spain; 8 Universidad Católica del Maule, Talca, Chile; 9 Department of Psychiatry, Centre Hospitalier Universitaire de Nîmes, Nîmes, Francia; 10 University College London, London, UK

**Keywords:** Schizophrenia, Psychosis

## Abstract

Poor insight in schizophrenia spectrum disorders (SSD) is linked with negative outcomes. This single-centre, assessor-blind, parallel-group 1-year follow-up randomised controlled trial (RCT) tested whether metacognitive training (MCT) (compared to psychoeducation) may improve insight and outcomes in outpatients with SSD assessed: at baseline (T0); after treatment (T1) and at 1-year follow-up (T2). Insight (primary outcome) was measured with (i) the Schedule for Assessment of Insight-Expanded version- (SAI-E), including illness recognition (IR), symptom relabelling (SR), treatment compliance (TC) and total insight scores (TIS); and (ii) the Beck Cognitive Insight Scale (BCIS). Between-group comparisons were nonsignificant, while within the MCT group (but not within controls) there was a significant medium effect size for improved TIS at T2 (*d* = 0.67, *P* = 0.02). Secondary outcomes included cognitive measures: Jumping to Conclusions (JTC), Theory of Mind (ToM), plus symptom severity and functioning. Compared to psychoeducation, MCT improved the PANSS excitement (*d* = 1.21, *P* = 0.01) and depressed (*d* = 0.76, *P* = 0.05) factors at T2; and a JTC task both at T1 (*P* = 0.016) and at T2 (*P* = 0.031). Participants in this RCT receiving MCT showed improved insight at 1-year follow-up, which was associated with better mood and reduced JTC cognitive bias. In this pilot study, no significant benefits on insight of MCT over psychoeducation were detected, which may have been due to insufficient power.

## Introduction

Insight (i.e., clinical insight) in schizophrenia spectrum disorders (SSD) has been linked with outcome—greater insight, better outcomes^1,2^. However, 50–80% of patients with SSDs^3^, particularly schizophrenia^4^, lack insight from first presentation^5^.

Thirty years ago clinical insight was proposed to be a multidimensional phenomenon encompassing (i) illness awareness, i.e., recognition of having a mental illness, (ii) symptom relabelling, defined as the ability to recall unusual mental events (e.g., hallucinations) as abnormal and (iii) treatment compliance^6^. This multidimensional model of clinical insight has been supported by three decades of research^7^. More specifically, independent first-episode psychosis (FEP) samples^8,9^ have replicated the David’s three-dimension model of clinical insight^6^. Interventions for improving clinical insight, including psychoeducation, psychoanalytically oriented therapies, cognitive-behavioural therapy (CBT), video-recorded self-observation and antipsychotics, have been minimally effective to date^10,11^, although metacognitive interventions revealed more promising results^12^.

Metacognition, defined as ‘knowledge and cognition about cognitive phenomena’^13^ or ‘the ability to think of one’s own and others’ thinking’^14^, has received much attention from research over the past few years. Specifically, metacognitive deficits have been consistently reported in SSDs^15^, and have been linked with a lack of clinical insight^7,16^. Of note, clinical insight, as detailed above, should be distinguished from the broader construct, cognitive insight, a metacognitive domain which includes the ability to evaluate and correct one’s distorted beliefs and misinterpretations (self-reflectiveness) and the tendency to overconfidence in one’s conclusions (self-certainty)^15,17^. Perhaps surprisingly, the relationship between cognitive and clinical insight has been found to be somewhat weak^17^.

In 2007, metacognitive training (MCT) was developed in Germany by Steffen Moritz and Todd Woodward. MCT seeks to plant the seeds of doubt by targeting cognitive biases leading to delusional thoughts rather than asking patients directly to talk about of their beliefs. MCT can be delivered individually or in group sessions by psychiatrists, psychologists, social workers, nurses and other therapists. The MCT manual consists of a PowerPoint presentation available at http://www.uke.de/mkt in thirty-seven languages free of charge, which includes ten Modules on different topics: Attributional Style (Module 1), Jumping to Conclusions (Modules 2 and 7), Changing Beliefs (Module 3), Empathy (Modules 4 and 6), Memory (Module 5), Depression and Self-Esteem (Modules 8 and 9) and Stigma (Module 10).

Although not consistently^18,19^, MCT was demonstrated to reduce positive^20–25^ and negative^26^ psychotic symptoms severity, cognitive biases, self-esteem and functioning^26^. However, evidence supporting the effects of specific MCT modules is limited^27^. Given the heterogeneity of delusional experiences in schizophrenia phenomenology^28^ and the MCT mechanism of action, namely inducing some doubt about the generation of such delusional ideas^29,30^, it would be interesting to know the effects of particular modules of MCT on specific delusion types^28^.

Of relevance, two core treatment targets of MCT, namely *Jumping to Conclusions* (JTC) cognitive bias and *Theory of Mind* (ToM) reasoning, have been linked with clinical insight^31^. Hence, MCT may improve insight via addressing cognitive insight, JTC and/or ToM. JTC, i.e., reaching a conclusion based on incomplete evidence, can be considered as a decision-making style common in psychosis^32–34^ which has been linked with delusions^35^ and poor clinical insight^31^. ToM can be defined as ‘the ability to attribute mental states— beliefs, intents, desires, emotions and knowledge—primarily to others’^36^. ToM deficits have been linked to lack of clinical insight^31^, consistently reported in patients with psychosis from first presentation^28^ associated with paranoia in schizophrenia, and finally, likely involve specific brain regions and pathways^37^. Although MCT targets other cognitive biases leading to delusional ideas, such as Bias Against Disconfirmatory Evidence (BADE), we decided to focus on JTC and ToM based on previous literature linking JTC and ToM (but not BADE) with clinical insight^31^.

Most importantly, only five previous short-term (over 6 months) randomised controlled trials (RCTs) using early-onset psychosis patients samples investigated the MCT effects on clinical insight^38–42^, with relatively modest results. To our knowledge, no previous long-term MCT RCT has examined the effects on cognitive and clinical insight changes in non-first-episode schizophrenia patients, including clinical and social outcomes^12^.

This RCT aimed to investigate whether MCT may improve clinical and cognitive insight (as co-primary outcomes) in outpatients with SSD over a 1-year follow-up. Secondary outcomes included JTC, ToM, symptomatic severity and psychosocial functioning. Compared with controls (an active psychoeducation group) we hypothesised that MCT will result in: (i) greater cognitive and clinical insight levels; (ii) an improvement in JTC cognitive bias and ToM performance, reduced symptom severity and better functioning, and (iii) we sought to explore whether these effects would persist at post treatment and at 1-year follow-up.

## Results

**Fig. 1:**
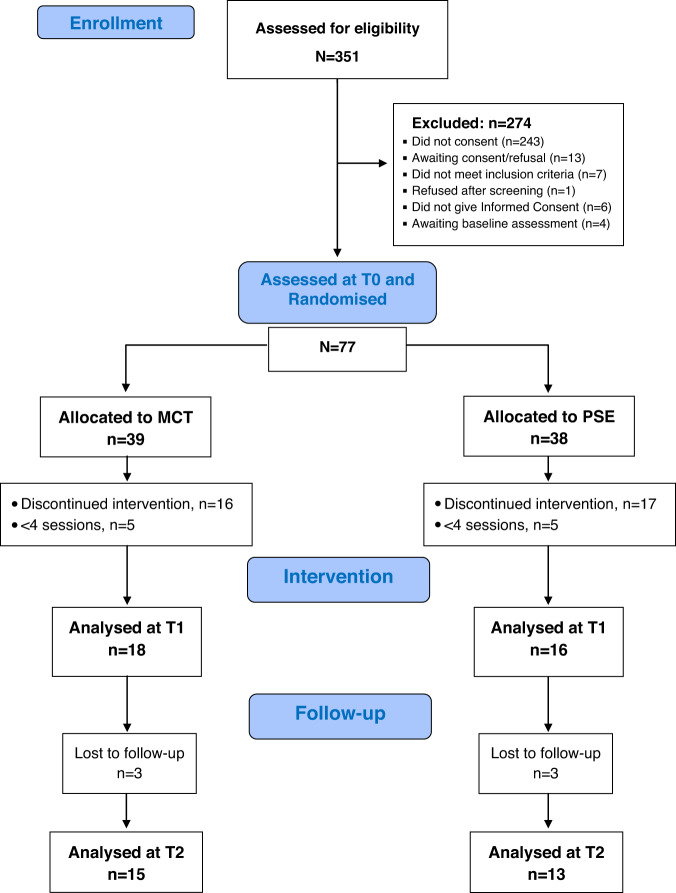
CONSORT chart of participant flow over the trial period. Completion rates and categorisation of reasons for discontinuation over the 1-year follow-up duration of the trial.

Figure 1 shows the CONSORT flow diagram of participants over the trial period. *N* = 77 individuals were assessed at T0 and randomised. *n* = 34 subjects (44.15%), who attended at least four sessions (e.g., ref. ^43^), were assessed and analysed at T1. At T2, *n* = 28 subjects were available and analysed. There were no between-group differences at any assessment. Of *n* = 34, subjects available at T1, 50% of them (median) attended 6 sessions (mean = 5.8 ± 2.4), with no between-group differences.

### Sample characteristics

There were no baseline differences in sociodemographic, clinical, premorbid adjustment, neurocognitive, psychopathological, insight, metacognitive and functioning variables between MCT and controls psychoeducation groups (Table 1), except that there was a significantly (*P* = 0.021) higher proportion of unmarried people in the MCT group (*n* = 35, 89.7%) than in controls (*n* = 26, 68.4%).

**Table 1:** Baseline sample characteristics (*N* = 77).

	MCT (*n* = 39)	PSE (*n* = 38)	Statistic	*P*
Sociodemographic variables				
Age (years)	45.6 ± 9.9	49.8 ± 9.3	*t*_75_ = −1.90	0.062
Gender (males)	21 (53.8)	20 (52.6)	*X*^2^_1_ = 0.01	0.91
Education level (primary)	6 (15.4)	7 (18.4)	*X*^2^_1_ = 0.13	0.72
Marital status (unmarried)	35 (89.7)	26 (68.4)	*X*^2^_1_ = 5.32	0.021
Employment status (unemployed)	28 (71.8)	28 (73.7)	*X*^2^_1_ = 0.03	0.85
Living status (alone)	4 (10.2)	4 (10.5)	*X*^2^_1_ = 0.00	0.97
Premorbid adjustment (PAS)				
Childhood	6.6 ± 4.6	5.0 ± 2.5	*t*_58.66_ = 1.92	0.060
Early adolescence	8.5 ± 5.3	6.7 ± 3.6	*t*_74_ = 1.75	0.085
Late adolescence	8.5 ± 5.6	6.9 ± 4.0	*t*_61.30_ = 1.40	0.17
Clinical variables				
Diagnosis (schizophrenia)	23 (58.9)	25 (65.8)	*X*^2^_1_ = 0.38	0.54
Duration of illness (>5 years)	33 (84.6)	36 (94.7)	*X*^2^_1_ = 2.12	0.15
Previous admissions	2.8 ± 2.8	4.1 ± 4.9	*t*_74_ = −1.45	0.15
Previous suicidal behaviour	17 (43.6)	14 (36.8)	*X*^2^_1_ = 0.36	0.55
Antipsychotics-related variables				
Monotherapy	23 (58.9)	22 (57.9)	*X*^2^_1_ = 0.01	0.92
Long-acting injections	31 (79.5)	21 (55.3)	*X*^2^_1_ = 5.15	0.02
Clozapine	6 (15.4)	6 (15.8)	*X*^2^_1_ = 0.00	0.96
Chlorpromazine equivalents	442.3 ± 310.0	461.2 ± 387.1	*t*_75_ = −0.24	0.81
Neurocognition				
IQ	104.7 ± 11.8	104.5 ± 11.7	*t*_75_ = 0.10	0.95
TMT B-A	69.0 ± 40.1	68.8 ± 47.8	*t*_69_ = 0.03	0.98
*Co-primary outcomes*				
Clinical insight (SAI-E)				
Illness recognition	5.5 ± 2.6	5.2 ± 2.8	*t*_75_ = 0.49	0.62
Symptoms relabelling	5.9 ± 2.8	5.8 ± 2.8	*t*_75_ = 0.09	0.93
Treatment compliance	4.3 ± 1.4	4.3 ± 1.7	*t*_75_ = −0.02	0.98
Total insight	15.7 ± 5.1	15.4 ± 5.5	*t*_75_ = 0.29	0.77
Cognitive insight (BCIS)				
Self-reflectiveness	16.3 ± 5.4	14.5 ± 4.6	*t*_72_ = 1.50	0.14
Self-certainty	7.4 ± 3.7	7.9 ± 3.1	*t*_71_ = −0.60	0.55
Composite index	9.0 ± 7.5	6.4 ± 5.4	*t*_68_ = 1.65	0.10
*Secondary outcomes*				
Symptomatic severity				
PANSS—Positive	8.6 ± 3.7	8.3 ± 3.7	*t*_75_ = 0.29	0.77
PANSS—Negative	14.3 ± 5.8	15.5 ± 6.0	*t*_75_ = −0.94	0.35
PANSS—Disorganisation	5.8 ± 2.8	6.3 ± 2.4	*t*_75_ = −0.70	0.49
PANSS—Mania	6.5 ± 1.7	6.0 ± 2.0	*t*_75_ = 1.15	0.25
PANSS—Depression	7.4 ± 2.6	6.5 ± 2.8	*t*_75_ = 1.41	0.16
CDSS—Total	3.6 ± 3.5	3.3 ± 4.2	*t*_75_ = 0.34	0.74
Jumping to Conclusions (JTC)				
JTC_85:15	23 (58.9)	19 (50.0)	*X*^2^_1_ = 1.12	0.29
JTC_60:40	17 (43.5)	21 (55.2)	*X*^2^_1_ = 0.86	0.35
Theory of Mind (ToM)				
Hinting Task	2.3 ± 1.2	2.2 ± 1.4	*t*_75_ = 0.40	0.69
ERTF	16.5 ± 2.0	17.2 ± 2.3	*t*_75_ = −1.54	0.13
Functioning				
GAF	62.4 ± 7.8	61.2 ± 7.1	*t*_75_ = 0.75	0.46
WHODAS	14.5 ± 9.5	15.8 ± 11.4	*t*_74_ = −0.56	0.57
SLDS	81.4 ± 10.4	78.8 ± 12.5	*t*_70_ = 0.97	0.34

*MCT* Metacognitive Training, *PSE* Psychoeducation, *PAS* Premorbid Adjustment Scale (Cannon-Spoor et al.^96^), *SAI-E* Schedule for Assessment of Insight, Expanded Version (Kemp & David^78^), *BCIS* Beck Cognitive Insight Scale (Beck et al.^15^), *PANSS* Positive and Negative Syndrome Scale for Schizophrenia (Kay et al.^86^), *CDSS* Calgary Depression Scale for Schizophrenia (Addington et al.^59^), *ERTF* Emotions Recognition Test Faces (Baron-Cohen et al.^51^), *GAF* General Assessment of Functioning (Endicott et al.^92^), *WHODAS* World Health Organization Disability Schedule (Üstün^93^), *SLDS* Satisfaction Life Domains Scale (Carlson et al.^94^).

Between-group differences in nominal (*X*^2^ test) and continuous (Student’s *t* test) are presented.

As noted above, only those individuals who attended four treatment sessions were analysed. In the MCT group no relevant differences between those who attended at least four sessions and those who did not were found (see Supplementary Table S1) except for one insight dimension, namely symptom relabelling (4.8 ± 2.3 vs. 6.9 ± 2.9, *t*_37_ = −2.44, *P* = 0.019). However, in the Psychoeducation control group (Supplementary Table S2), attendees (compared with non-attendees) had higher IQ (109.7 ± 12.0 vs. 100.7 ± 10.1, *t*_36_ = 2.50, *P* = 0.017) and BCIS Composite Index (8.9 ± 5.1 vs. 4.5 ± 5.0, *t*_32_ = 2.51, *P* = 0.017), better functioning in terms of WHODAS (21.3 ± 12.9 vs. 12.1 ± 8.7, *t*_35_ = 2.61, *P* = 0.013) and SLDS (72.9 ± 12.1 vs. 83.6 ± 10.8, *t*_34_ = −2.81, *P* = 0.008) and they were less likely to have JTC as assessed by the 85:15 Beads Task (*n* = 4, 25.0% vs. *n* = 15, 68.2%, *X*^2^_1_ = 6.91, *P* = 0.009) (Supplementary Table S2). Most importantly, for those who attended at least 4 treatment sessions (*n* = 34) no significant baseline differences between MCT (*n* = 18) and psychoeducation (*n* = 16) groups were found except for WHODAS (*P* = 0.047) and SLDS (*P* = 0.034) total scores, none of which were the primary outcomes of the study (Supplementary Table S2). Analyses were not therefore controlled for baseline data.

### Between-group differences in outcomes

Table 2 presents between-group differences in continuous outcome measures after treatment and at follow-up. Of note, the only nominal non-continuous variable was JTC.

**Table 2:** Between-Group differences in continuous outcome measures after treatment (T1) and at 1-year follow-up (T2) vs. baseline (T0).

	MCT			PSE			Between-group differences			
	T0 *N* = 18	T1 *N* = 18	T2 *N* = 15	T0 *N* = 16	T1 *N* = 16	T2 *N* = 13	T0 vs. T1		T0 vs. T2	
	Mean (SD)	Mean (SD)	Mean (SD)	Mean (SD)	Mean (SD)	Mean (SD)	*d*	*P*	*d*	*P*
*Co-primary outcomes*										
Clinical insight (SAI-E)										
Illness recognition	5.39 (2.50)	5.61 (2.48)	5.73 (2.91)	6.00 (2.99)	5.87 (2.87)	6.23 (2.77)	0.21	0.55	0.21	0.58
Symptom relabelling	4.78 (2.26)	5.94 (3.17)	6.13 (2.42)	6.00 (1.32)	5.56 (2.47)	6.38 (3.15)	0.56	0.11	0.23	0.56
Treatment compliance	4.50 (1.54)	4.50 (1.58)	5.13 (1.19)	4.88 (1.54)	4.62 (1.54)	4.92 (1.19)	0.15	0.65	0.39	0.31
Total insight	14.67 (4.87)	16.05 (5.80)	17.00 (4.97)	16.88 (4.69)	16.06 (5.13)	17.53 (4.86)	0.52	0.13	0.21	0.58
Cognitive insight (BCIS)										
Self-reflectiveness	16.11 (5.21)	16.89 (5.29)	17.00 (5.41)	15.63 (4.67)	15.56 (4.02)	15.92 (3.37)	0.21	0.54	0.19	0.61
Self-certainty	7.24 (3.49)	7.28 (4.38)	7.27 (3.71)	6.73 (3.06)	6.81 (3.45)	7.46 (3.73)	−0.10	0.77	−0.14	0.70
Composite index	9.12 (7.92)	9.61 (8.48)	9.73 (8.38)	8.87 (5.14)	8.75 (4.93)	9.08 (3.89)	0.29	0.40	0.15	0.69
*Secondary outcomes*										
Symptomatic severity										
PANSS—Positive	7.72 (2.70)	8.11 (3.80)	7.33 (2.16)	9.20 (3.87)	7.50 (3.14)	7.69 (3.22)	0.69	0.05	−0.03	0.94
PANSS—Negative	12.89 (5.99)	14.61 (6.63)	13.67 (5.45)	16.00 (5.12)	17.38 (5.49)	17.38 (2.90)	0.06	0.87	−0.31	0.44
PANSS—Disorg.	5.22 (3.19)	5.50 (2.26)	5.47 (2.42)	6.25 (2.21)	6.75 (2.08)	6.85 (2.07)	−0.09	0.78	0.13	0.74
PANSS—Mania	6.39 (2.19)	6.39 (3.22)	6.20 (2.43)	5.69 (1.99)	5.75 (1.84)	8.08 (2.46)	−0.02	0.94	−1.21	0.01
PANSS—Depression	7.94 (2.88)	6.11 (3.01)	5.73 (2.46)	6.81 (3.10)	6.88 (3.01)	6.69 (2.78)	−0.65	0.07	−0.76	0.05
CDSS (total)	3.50 (3.87)	3.11 (3.53)	2.40 (3.29)	4.69 (4.03)	4.69 (4.76)	5.08 (4.96)	−0.15	0.68	−0.53	0.16
Theory of Mind										
ERTF	16.56 (2.20)	16.89 (1.87)	17.27 (1.49)	17.31 (2.30)	18.00 (2.00)	17.46 (1.90)	−0.20	0.57	0.09	0.81
Hinting Task	2.33 (1.41)	2.22 (1.35)	2.53 (0.99)	2.38 (1.45)	2.13 (1.50)	2.46 (0.97)	0.09	0.79	−0.15	0.70
Functioning										
GAF	64.56 (9.51)	62.06 (9.86)	61.47 (10.71)	60.88 (7.85)	59.94 (8.70)	58.46 (7.48)	−0.18	0.59	−0.18	0.63
WHODAS	13.89 (7.47)	14.61 (10.60)	12.40 (9.80)	21.33 (12.93)	22.00 (13.97)	19.31 (10.63)	−0.11	0.74	−0.07	0.85
SLDS	81.71 (10.81)	84.00 (13.32)	87.36 (9.18)	72.88 (12.07)	74.69 (14.64)	79.31 (14.21)	0.09	0.79	0.05	0.91

*MCT* Metacognitive Training, *PSE* Psychoeducation, *d* Cohen’s *d* (effect size), *SAI-E* Schedule for Assessment of Insight, Expanded Version (Kemp & David^78^), *BCIS* Beck Cognitive Insight Scale (Beck et al.^15^), *PANSS* Positive and Negative Syndrome Scale for Schizophrenia (Kay et al.^86^), *CDSS* Calgary Depression Scale for Schizophrenia (Addington et al.^59^), *ERTF* Emotions Recognition Test Faces (Baron-Cohen et al.^51^), *GAF* General Assessment of Functioning (Endicott et al.^92^), *WHODAS* World Health Organization Disability Schedule (Üstün^93^), *SLDS* Satisfaction Life Domains Scale (Carlson et al.^94^).

Student’s *t* test examined between-group differences in presented continuous variables changes from T0 to T1 and from T0 to T2, which are reported in the right four columns as effect sizes, namely Cohen’s *d* and the corresponding *P* value.

#### Primary outcomes

Although the MCT group SAI-E scores improved from T0 to T1 and from T0 to T2, between-arm comparisons yielded nonsignificant effect sizes (Table 2).

#### Secondary outcomes

With regard to the ‘easier’ JTC 85:15 Task, in the MCT group, there was a decrease in the number of subjects with JTC from T0 (*n* = 9, 50%) to T1 (*n* = 6, 33.3%) (*P* = 0.25), while at T2 only *n* = 4 subjects (26.7%) jumped to conclusions (*P* = 0.22). In the psychoeducation group, there was an increase in the number of individuals who jumped to conclusions from T0 (*n* = 4, 25%) to T1 (*n* = 6, 37.5%) (*P* = 0.62), although at T2 only *n* = 5 subjects (38.4%) had JTC (*P* = 0.99).

Regarding the JTC 60:40 Task, in the MCT group there was a significant decrease in the number of subjects who jumped to conclusions from T0 (*n* = 9, 50%) to T1 (*n* = 2, 11.1%) (*P* = 0.016) and from T0 (*n* = 9, 50%) to T2 (*n* = 2, 11.1%) (*P* = 0.031), while in the psychoeducation group differences were nonsignificant: *n* = 5 (27.8%) both at T0 and at T1 (*P* = 0.99) and *n* = 2 (12.5%) (*P* = 0.37) at T2.

No small or significant effects on ToM tasks were found (Table 2).

With regard to positive and negative psychotic symptoms and disorganisation, effect sizes did not reach significance, although MCT was superior to psychoeducation at an almost significant large effect size in terms of depression (*d* = 0.76, *P* = 0.05), which was significant for the PANSS excitement factor (*d* = 1.21, *P* = 0.01), at T2.

In terms of functioning, effect sizes were nonsignificant (Table 2).

**Fig. 2:**
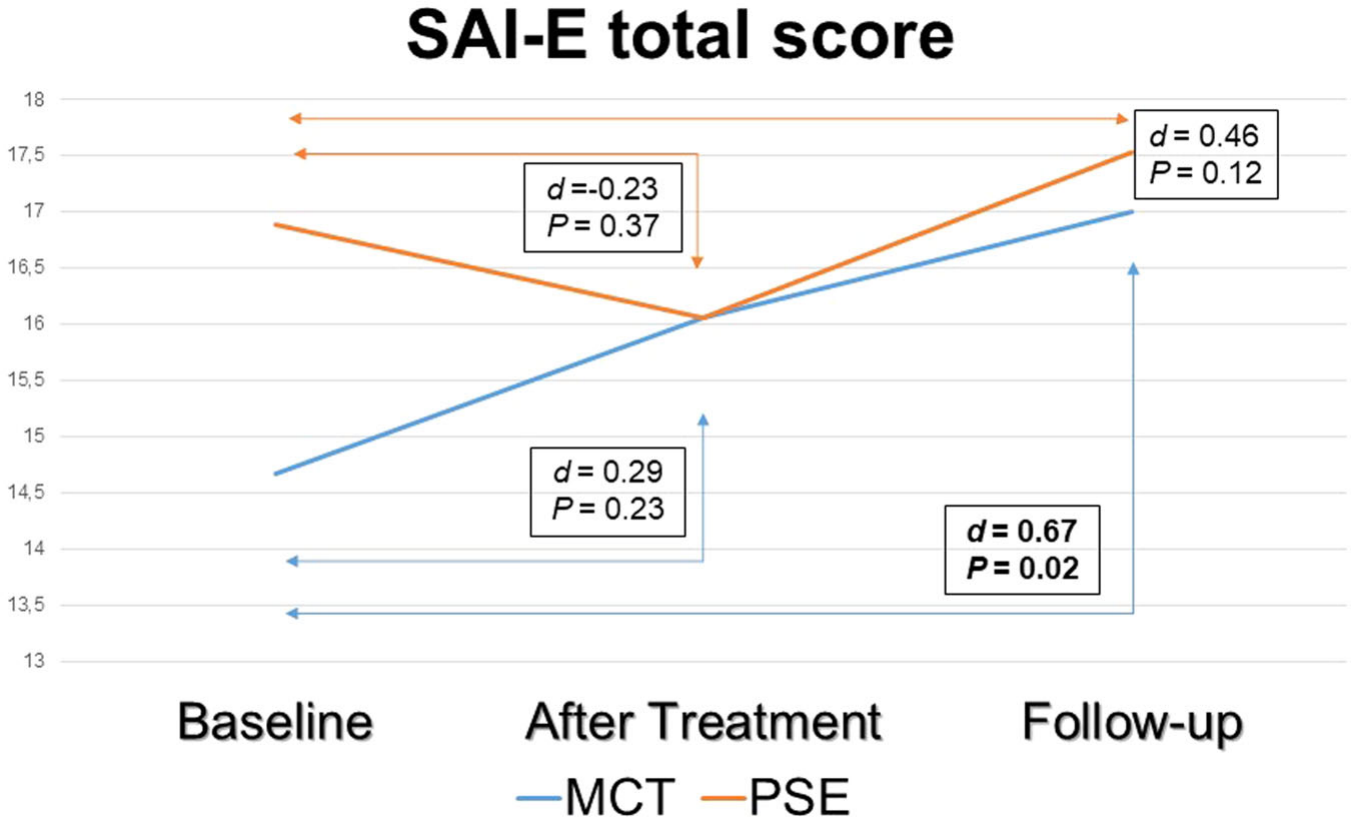
Between-group differences in SAI-E total score changes over the 1-year follow-up. Within each treatment group, Student’s *t* test compared SAI-E total scores changes from baseline to post treatment and from baseline to 1-year follow-up. The blue line indicates values changes for the MCT group, while the orange line indicates values changes for the psychoeducation group. The text boxes report the effect sizes as Cohen’s *d* coefficient and its *P* value.

**Fig. 3:**
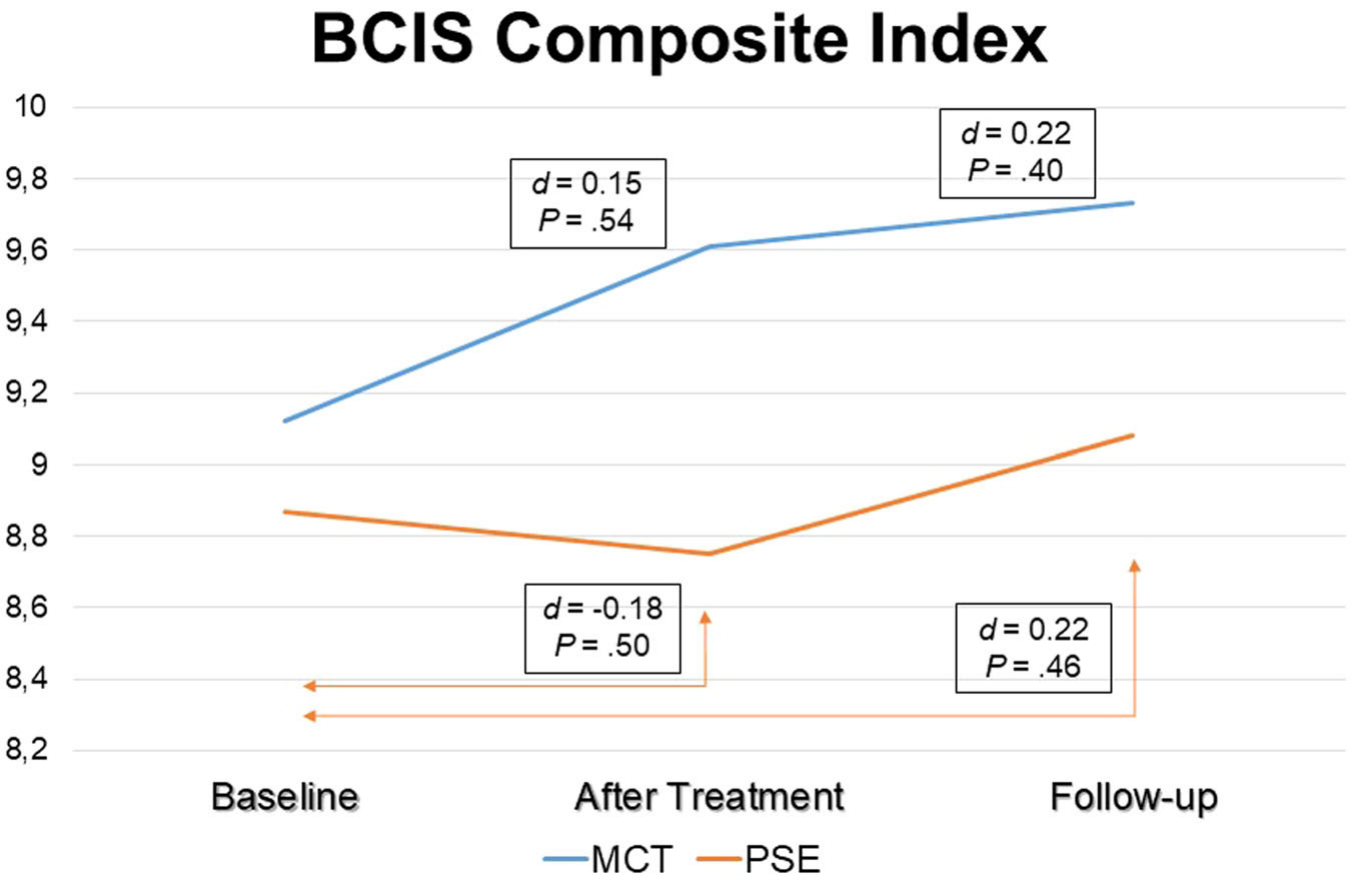
Between-group differences in BCIS Composite Index changes over the 1-year follow-up. Within each treatment group, Student’s *t* test compared BCIS Composite Index changes from baseline to post treatment and from baseline to 1-year follow-up. The blue line indicates values changes for the MCT group, while the orange line indicates values changes for the psychoeducation group. The text boxes report the effect sizes as Cohen’s *d* coefficient and its *P* value.

Between-treatment differences in insight changes over the trial period are also plotted in Fig. 2 (SAI-E total score, i.e., clinical insight) and in Fig. 3 (BCIS Composite Index, i.e., cognitive insight).

### Within-group differences in outcome measures

In terms of insight gain in the MCT group at T2 we found a significant medium effect size for TIS (*d* = 0.67, *P* = 0.02) and almost significant effect sizes for SR (*d* = 0.55, *P* = 0.05) and for TC (*d* = 0.52, *P* = 0.06) at T2, which was not replicated at T1. In the psychoeducation group, all effect sizes for insight changes at T1 and at T2 were nonsignificant.

**Fig. 4:**
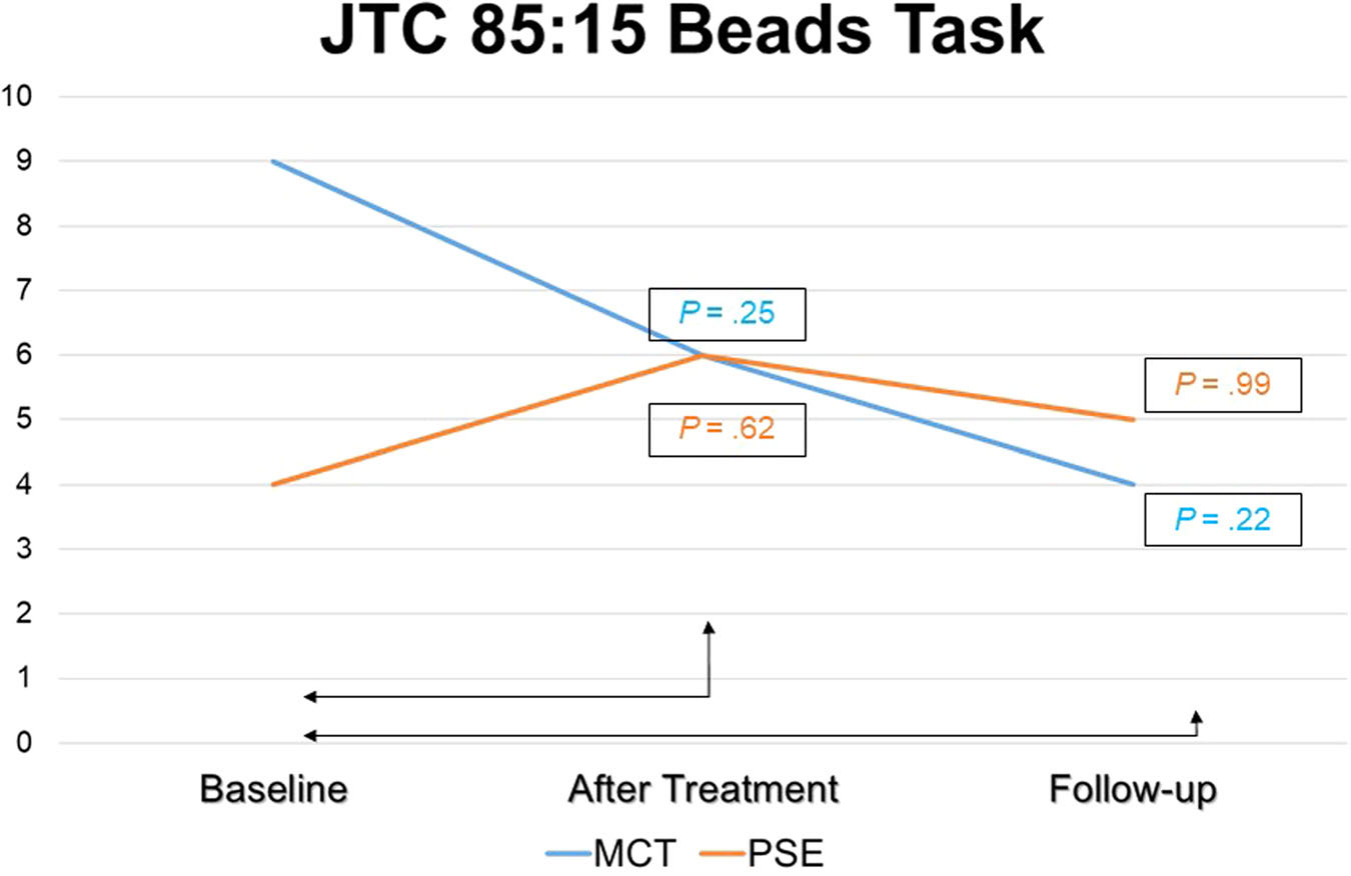
Jumping to Conclusions 85:15 Task. Number of patients jumping to conclusions in the 85:15 Beads Task at each assessment (at baseline, at post treatment and at follow-up).

**Fig. 5:**
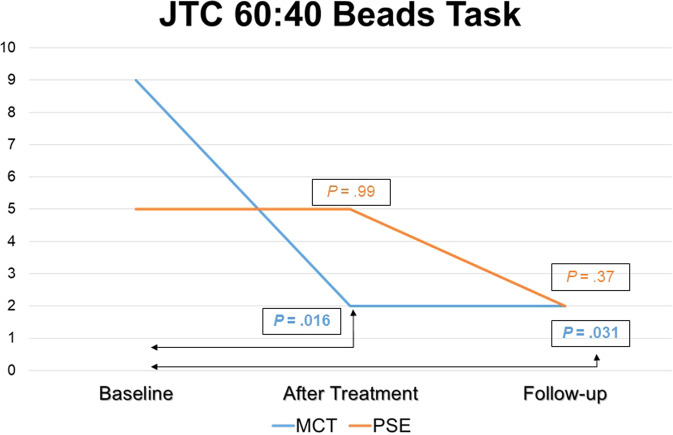
Jumping to Conclusions 60:40 Task. Number of patients jumping to conclusions in the 60:40 Beads Task at each assessment (at baseline, at post treatment and at follow-up).

Further within-group differences in outcomes are shown in Table 3 (MCT group) and in Table 4 (Controls). Also, within-group differences in the JTC Beads Tasks over the trial period are plotted in Fig. 4 (85:15) and Fig. 5 (60:40), below.

**Table 3:** Within-MCT group differences in continuous outcome measures after treatment (T1) and at follow-up (T2) compared with baseline (T0).

	T0 vs. T1					T0 vs. T2				
	Mean diff	*df*	*t*	*d*	*P*	Mean diff	*df*	*t*	*d*	*P*
*Co-primary outcomes*										
Clinical insight (SAI-E)										
Illness recognition	0.22	17	0.58	0.14	0.57	0.53	14	1.14	0.30	0.27
Symptom relabelling	1.17	17	1.57	0.37	0.13	1.47	14	2.13	0.55	0.05
Treatment compliance	0	17	0	0	1	0.93	14	2.02	0.52	0.06
INSIGHT	1.39	17	1.25	0.29	0.23	2.93	14	2.60	0.67	0.02
Cognitive insight (BCIS)										
Self-reflectiveness	0.78	17	0.72	0.17	0.48	1.47	14	1.10	0.28	0.29
Self-certainty	−0.06	16	−0.09	−0.02	0.93	−0.33	14	−0.32	−0.08	0.76
Composite iIndex	0.94	16	0.62	0.15	0.54	1.80	14	0.86	0.22	0.40
*Secondary outcomes*										
Symptomatic severity										
PANSS—Positive	0.39	17	0.53	0.13	0.60	−1.00	14	−1.51	−0.39	0.15
PANSS—Negative	1.72	17	1.27	0.30	0.22	0.87	14	1.08	0.28	0.30
PANSS—Disorg.	0.28	17	0.50	0.12	0.62	0.80	14	2.17	0.56	0.047
PANSS—Mania	0	17	0	0	1	−0.33	14	−0.67	−0.17	0.51
PANSS—Depression	−1.83	17	−2.67	−0.63	0.016	−2.00	14	−2.56	−0.66	0.02
CDSS (total)	−0.39	17	−0.82	−0.19	0.42	−0.67	14	−1.00	–0.26	0.33
Theory of Mind (ToM)										
ERTF	0.33	17	0.75	0.18	0.46	0.53	14	1.00	0.26	0.33
Hinting Task	−0.11	17	−0.30	−0.07	0.77	−0.13	14	−0.33	−0.09	0.74
Functioning										
GAF	−2.50	17	−1.06	−0.25	0.30	−4.13	14	−1.74	−0.45	0.10
WHODAS	0.72	17	0.26	0.06	0.80	−0.67	14	−0.19	−0.05	0.85
SLDS	2.71	16	1.14	0.28	0.27	5.38	12	1.77	0.49	0.10

*MCT* Metacognitive Training, *PSE* Psychoeducation, *d* Cohen’s *d* (effect size), *SAI-E* Schedule for Assessment of Insight, Expanded Version (Kemp & David^78^), *BCIS* Beck Cognitive Insight Scale (Beck et al.^15^), *PANSS* Positive and Negative Syndrome Scale for Schizophrenia (Kay et al.^86^), *CDSS* Calgary Depression Scale for Schizophrenia (Addington et al.^59^), *ERTF* Emotions Recognition Test Faces (Baron-Cohen et al.^51^), *GAF* General Assessment of Functioning (Endicott et al.^92^), *WHODAS* World Health Organization Disability Schedule (Üstün^93^), *SLDS* Satisfaction Life Domains Scale (Carlson et al.^94^).

Student’s *t* test examined within-group differences in continuous variables changes from T0 to T1 and from T0 to T2, which are reported as effect sizes, namely Cohen’s *d* coefficient and the corresponding *P* value.

**Table 4:** Within-Psychoeducation group differences in continuous outcome measures after treatment (T1) and at follow-up (T2) compared with baseline (T0).

	T0 vs. T1					T0 vs. T2				
	Mean diff.	gf	*t*	*d*	*P*	Mean diff.	gf	*t*	*d*	*P*
*Co-primary outcomes*										
Clinical insight (SAI-E)										
Illness recognition	−0.12	15	−0.29	−0.07	0.77	0.92	12	1.76	0.49	0.10
Symptom relabelling	−0.44	15	0.71	−0.18	0.49	0.77	12	0.79	0.22	0.44
Treatment compliance	−0.25	15	−0.74	−0.19	0.47	0.31	12	0.80	0.22	0.44
INSIGHT	−0.81	15	−0.92	−0.23	0.37	2	12	1.67	0.46	0.12
Cognitive insight (BCIS)										
Self-reflectiveness	−0.06	15	−0.07	−0.02	0.94	0.58	12	0.54	0.16	0.60
Self-certainty	0.20	14	0.36	0.09	0.72	0.15	13	0.23	0.06	0.82
Composite index	−0.47	14	0.69	−0.18	0.50	0.83	12	0.77	0.22	0.46
*Secondary outcomes*										
Symptomatic severity										
PANSS—Positive	−1.69	15	−2.29	−0.57	0.036	−0.92	12	−1.16	−0.32	0.27
PANSS—Negative	1.38	15	0.91	0.23	0.38	2.31	12	1.41	0.39	0.18
PANSS—Disorg.	0.50	15	0.85	0.21	0.41	0.54	12	0.79	0.22	0.45
PANSS—Mania	0.06	15	0.10	0.03	0.92	2.46	12	3.29	0.91	0.006
PANSS—Depression	0.06	15	0.08	0.02	0.93	0.08	12	0.12	0.03	0.91
CDSS (total)	0	15	0	0	1	0.54	12	1.07	0.30	0.30
Theory of Mind (ToM)										
ERTF	0.69	15	1.58	0.40	0.13	0.31	12	0.39	0.11	0.70
Hinting Task	−0.25	15	−0.69	−0.17	0.50	0.08	12	0.21	0.06	0.84
Functioning										
GAF	−0.94	15	−0.55	−0.14	0.59	−2.69	12	−1.55	−0.43	0.15
WHODAS	1.87	14	0.95	0.24	0.36	0.08	11	0.04	0.01	0.97
SLDS	1.81	15	0.80	0.20	0.43	4.92	12	1.89	0.52	0.083

*MCT* Metacognitive Training, *PSE* Psychoeducation, *d* Cohen’s *d* (effect size), *SAI-E* Schedule for Assessment of Insight, Expanded Version (Kemp & David^78^), *BCIS* Beck Cognitive Insight Scale (Beck et al.^15^), *PANSS* Positive and Negative Syndrome Scale for Schizophrenia (Kay et al.^86^), *CDSS* Calgary Depression Scale for Schizophrenia (Addington et al.^59^), *ERTF* Emotions Recognition Test Faces (Baron-Cohen et al.^51^), *GAF* General Assessment of Functioning (Endicott et al.^92^), *WHODAS* World Health Organization Disability Schedule (Üstün^93^), *SLDS* Satisfaction Life Domains Scale (Carlson et al.^94^).

Student’s *t* test examined within-group differences in continuous variables changes from T0 to T1 and from T0 to T2, which are reported as effect sizes, namely Cohen’s *d* coefficient and the corresponding *P* value.

## Discussion

### Principal findings

We carried out a pilot study to test whether metacognitive training (MCT) (compared with psychoeducation) may improve clinical and cognitive insight in an unselected sample of outpatients with SSD in order to path the way for a larger trial. As secondary outcomes, we looked at decision-making and mentalizing, that is, JTC cognitive bias and ToM tasks, respectively, symptoms severity and psychosocial functioning.

Results provided some support for our first hypothesis, which predicted that (compared to psychoeducation) MCT would result in greater clinical and cognitive insight gain. In particular, we found MCT to improve both clinical and cognitive insight, although MCT did not show significant benefits compared to psychoeducation, which may have been due to insufficient statistical power. However, within-group comparisons revealed that while the MCT effect on total clinical insight scores changes at 1-year follow-up reached significance, psychoeducation failed to do this, somewhat consistent with our first hypothesis. This said, given the *pilot* nature of the trial and its limited power, this finding should be taken very cautiously. Regarding secondary outcomes (compared to psychoeducation) MCT showed significant effects sizes on JCT and almost significant effects sizes on the excitement and depressed PANSS factors; however, no such effects were observed on ToM and functioning. These findings partially supported our second hypothesis, which postulated that MCT would result in an improvement in JTC cognitive bias and ToM performance, reduced symptom severity and better functioning. We also noted that most of these effects were larger after treatment than at a 1-year follow-up.

### Metacognitive training effects on clinical and cognitive insight

Not much progress has been made in treatments for (clinical) insight in psychosis^10,11^, although a very recent systematic review and meta-analysis^12^, which included five RCTs (*N* = 244) on MCT^38,39,41,42,44^, showed MCT to increase clinical insight at a larger effect than in controls. In this pilot study, however, between-group comparisons for all SAI-E scores failed to reveal MCT to be superior to psychoeducation, which may have been owing to the relatively small sample size as a result of the COVID-19 outbreak in the middle of the trial. In addition, we tested MCT against an active control intervention, which diminishes effect sizes in comparison with trials using TAU as comparator^45^.

Taking a multidimensional approach to clinical insight^6^, the larger effect sizes were observed for the symptom relabelling component, thus replicating some previous trials^38,39,44^. Therefore, MCT seems to be more useful in improving the ability to reframe the meaning of certain psychotic symptoms rather than in improving illness awareness as a whole or treatment compliance. Moreover, treatment compliance does not appear to have a metacognitive basis, hence being less amenable to metacognitive interventions^12^, although one trial^41^ conflicted with this notion.

Interestingly, we found larger effects immediately after treatment than at follow-up. Only three previous RCTs^38,42,44^ examined the effects of MCT on clinical insight at 6 months, two of which^38,42^ replicated this pattern. Future trials are warranted to compare whether adding MCT *maintenance* sessions may alter these results. Regretfully, we could not evaluate the impact of each MCT module on clinical insight changes due to limited statistical power. This said, the more relevant benefits for an individual clinical insight dimension, such as symptom relabelling, are likely to particularly reflect the benefits from two specific modules, namely attributional style (Module 1) and Changing Beliefs (Module 3), which warrants future investigation in a full-scale trial.

*Cognitive insight* was put forward by Beck and colleagues in 2004, who also validated a 15-item self-rated scale, the Beck Cognitive Insight Scale (BCIS) which yielded two factors, namely self-reflectiveness and self-certainty^15^. Therefore, interventions targeting cognitive insight aim to increase self-reflectiveness and to decrease self-certainty. In this respect, in contrast with our 2020 meta-analysis^12^ and recent FEP studies^27,46,47^, we found nonsignificant effect sizes when comparing MCT to psychoeducation, which may have been due to lack of power. Therefore, future trials with larger sample sizes and more prolonged follow-up periods are warranted to address this clinically relevant issue. More specifically, our results showing (non-significantly) greater effects for self-reflectiveness than for self-certainty appear to suggest that self-reflectiveness may be more amenable to group MCT than self-certainty, although this remains to be confirmed. Future studies may examine whether *individual* MCT-based interventions, such as a MCT-based smartphone application (https://clinical-neuropsychology.de/app_en)^48^ and individual face-to-face MCT sessions^38,49^ may reduce self-certainty levels at a larger effect size than group MCT. On the other hand, cognitive insight, especially self-certainty, may behave as a trait (rather than a state), which cannot be modified through intervention.

### Metacognitive training effects on JTC and ToM

We replicated the positive effect of MCT on JTC^46,50^. Indeed, MCT specifically addresses JTC in two modules^23^, although the potential influence of JTC cognitive bias on lack of insight in psychosis remains unknown.

Contrary to our expectations (hypothesis ii), MCT was not shown to improve ToM deficits when compared with psychoeducation. It could be argued, however, that the two ToM measures of the trial, namely the Emotions Recognition Test Faces^51,52^ activity and the Hinting Task^53^, may have failed to capture those ToM elements targeted by MCT^54^. Also, ToM deficits in *early* psychosis may be more prone to modification^28^ than in *later* stages of the illness. Certainly, mentalizing impairment appears to be a *trait* marker of schizophrenia^55^.

### Positive effects of metacognitive training on mood

MCT improved mood by reducing excitement and depressive symptoms severity, particularly at follow-up. In particular, it should be noted that both excitement and depressive symptoms were based on the PANSS factors^56^ which included the following PANSS items: *excitement*—excitement (P4), Hostility (P7), Uncooperativeness (G8) and Poor impulse control (G14)—and *depression*—Anxiety (G2), Guilt Feelings (G3) and Depression (G6). In particular, a recent meta-analysis of 63 studies across 22 countries demonstrated self-stigma to positively correlate with depressive symptoms in schizophrenia spectrum disorders—greater stigma, more severe depressive symptoms—while the correlation between self-stigma and self-esteem was negative—greater stigma, worse self-esteem-^57^. Hence, there are grounds to speculate that the above MCT-induced mood improvement could be explained, in part, by two MCT additional modules, namely Self-Esteem (Module 9) and Stigma (Module 10), which were later added to the original 8-module MCT package^58^. However, these results were not replicated with the Calgary Depression Scale for Schizophrenia (CDSS)^59^ total score, which could be attributable to the limited power of the trial. Future studies using different scales are therefore warranted.

Although two previous meta-analyses replicated the relationship between mood and clinical insight—lower mood, greater insight^60,61^—the causality direction remains far from clear. On the one hand, becoming aware of having a psychotic illness could be thought to lead to more severe depressive symptoms, which is known as the *demoralisation syndrome*^62^. On the other hand, depressed patients are subject to cognitive distortions which makes them more pessimistic about themselves, including illnesses—the depressive realism model^63^—hence scoring higher on insight scales at assessment. The so-called (clinical) Insight Paradox^64^ has also been replicated for cognitive insight, particularly for self-reflectiveness^65,66^ in SSD, which was found to mediate the impact of depression on general psychological distress^67^. However, the relationship between insight and increased suicide risk has not been confirmed^68,69^. Nonetheless, more theoretical debate and empirical research is needed to better understand the clinical meaning of depression in schizophrenia, thus improving patient outcomes^70^.

Of note, no baseline differences were found between those who attended four treatment sessions and those who did not except for the MCT group (see Supplementary Tables S1, S2 and S3 in the Supplementary online material). In particular, within the MCT group those who dropped out of the trial before the fourth session had greater baseline insight into mental symptoms than those who attended four or more sessions. Certainly, recalling mental events as pathological must be very distressful. Hence, those with greater ability to recall mental experiences as abnormal may be at a higher risk of disengagement from an intervention such as MCT, which seeks to encourage self-reflection on these phenomena.

### Strengths and limitations

This is the first 1-year follow-up RCT examining the effects of MCT on multiple dimensions of clinical and cognitive insight (as co-primary outcomes) measured with validated instruments (SAI-E and BCIS, respectively) in a sample of SSD outpatients. Participants were randomised to either MCT or psychoeducation and the same assessor (JDLM) blinded to the treatment allocation evaluated outcomes across assessments. MCT founders were uninvolved in the trial and participants did not receive a financial compensation. We also controlled for the effect of attending a weekly therapeutic group since controls received an active intervention (psychoeducation), which probably improved recruitment to the study and conferred some ethical benefits in comparison to studies with TAU comparators, although this may have diminished effect sizes favouring MCT^45^.

However, some limitations should be borne in mind when interpreting our pilot RCT results. First, recruitment and intervention groups had to be stopped in March 2020 owing to the COVID-19 outbreak in Spain. Not only did this reduce the study power, but also measures for combating COVID-19, such as prolonged confinement periods, may have had a negative impact on functioning-related outcomes at follow-up. Although unlikely given the consistency of results from this *pilot* study, a potential *‘regression to the mean’* phenomenon cannot be fully ruled out. Future large-scale trials are therefore required. Hence, the *true* effect size of MCT on insight changes remains to be established. In addition, given the aforementioned power issues we did not examine potential MCT effects on individual PANSS items, such as delusions, or the module-specific effects on insight. Also, we only analysed those who attended at least four treatment sessions, in line with previous studies^38,43^. Second, other variables such as antipsychotics^71^, which were not evaluated in this trial, may have affected our results. Third, although not evaluated in this study, the potential improvement in other cognitive processes targeted by MCT, such as Bias Against Disconfirmatory Evidence, may have contributed to MCT-related insight gain, which requires further investigation. Fourth, regretfully we did not conduct a satisfaction survey or feedback questionnaire^72^, although the high attendance rates and the lack of issues raised by attendees suggested high levels of satisfaction. We cannot rule out that between-arm differences in insight levels over the trial period may have contributed to attrition issues which may have affected the results, although this seems unlikely. Finally, these findings may not apply to other settings such as primary care and those living in rural areas. In addition, participants consented to a lengthy protocol, including three face-to-face assessments over one year, which may have excluded those individuals with poorer insight.

## Conclusions

This RCT was designed to compare MCT with psychoeducation in an unselected sample of outpatients with SSD with the aim of investigating effects on insight and some clinical and social outcomes. Regretfully, the COVID-19 outbreak in Spain in March 2020 prevented the trial from recruiting the required sample size, thus requiring us to reconsider the original RCT as a *pilot* study. Although much caution is therefore needed when interpreting the results, MCT proved useful in improving insight and some outcomes, such as JTC and mood, in this sample of SSD patients. Of note, conducting such a trial remains feasible since no adverse effects were observed and most participants remained clinically stable. These promising results therefore strongly justify a larger scale RCT and future research in this area.

## Methods

### Study design and randomisation process

Single-centre, assessor-blind, parallel-group, two-armed, 1-year follow-up RCT. After baseline (T0) assessment, participants were randomised to either group MCT (experimental intervention) or a psychoeducation group (controls) through a computerised algorithm independent of the investigators (no stratification factors) in blocks of 10 subjects (maximum number of each group) and assessor (JDLM)-patient blind. Participants were aware of the intervention so the RCT was not double-blind, as in most non-pharmacological trials. Reassessments took place after treatment (T1) and at 1-year follow-up (T2), which were carried out by the same assessor (JDLM) blind to the group allocation (*assessor-blind*).

### Sample and eligibility criteria

Participants came from the publicly-funded Hospital Universitario Fundación Jiménez Díaz (Madrid, Spain). Inclusion criteria were: (i) outpatient status; (ii) age: 18-64 years, both inclusive; and iii) diagnosis, namely SSD based on the Mini International Neuropsychiatric Interview, 5th Edition, (MINI)^73^, which included schizophrenia, schizoaffective disorder, delusional disorder and psychotic disorder Not Otherwise Specified, according to either International Statistical Classification of Diseases (ICD), 10th Revision^74^.

Recruitment began on the 06/17/2019 and had to be stopped on the 03/11/2020 due to the COVID-19 outbreak in Spain. Exclusion criteria were: (i) IQ ≤ 70, which was assessed with the short form of the Wechsler Adults Intelligence Scale (WAIS)-IV^75^, (ii) a history of head injury and/or a neurological condition; (iii) having received a metacognitive intervention within the previous year; (iv) low level of Spanish; (v) lack of cooperativeness for participating in the intervention groups detailed below, as judged by the treating consultant psychiatrist or psychologist. Participants provided written informed consent as approved by the Local Research Ethics Committee (EC044-19_FJD-HRJC). The RCT is registered at ClinicalTrials.gov (NCT04104347). Participants were not financially compensated for completing the assessments and/or receiving the interventions. The full study protocol of this RCT was published elsewhere^76^.

### Variables

#### Co-primary outcomes

*Clinical insight* was assessed with the Spanish version^77^ of the Schedule for Assessment of Insight, expanded version (SAI-E)^78^, which provides scores on three insight dimensions based on David’s model of insight^6^—illness recognition (IR), symptom relabelling (SR), treatment compliance (TC)—and a total insight score (TIS). The scale was found to be easily applicable in routine clinical practice^79^ and good to excellent inter-rater reliability was reported, with total insight scores intra-class correlations coefficients ranging from 0.92 to 0.98 (*P* < 0.001)^8^. JDLM was trained by the author scale (ASD) and they both co-led the validation study of the SAI-E Spanish version^77^, which was used in this RCT.

*Cognitive insight* was evaluated by the Spanish version^80^ of the Beck Cognitive Insight Scale (BCIS)^15^. The BCIS is a 15-item self-administered scale which includes 9 items assessing self-reflectiveness and 6 items enquiring about self-certainty. A composite index can thus be calculated by subtracting self-certainty from self-reflectiveness. Internal consistency was found to be acceptable, with Cronbach’s α ranging from 0.60 to 0.68 across individual BCIS items^80^.

#### Secondary outcomes

Secondary outcomes included Jumping to Conclusions (JTC) and Theory of Mind (ToM), symptomatic severity and functioning.

*Jumping to Conclusions (JTC)* was measured with a computerised version of the *Beads Task*^81^. Participants are shown two jars containing coloured beads in different, although reciprocal, proportions. On the basis of probability (in task 1 the probability is 85:15, while in task 2 the probability is 60:40), the individual must decide the jar to which the extracted bead belongs. *JTC* was rated as present/absent based on the ‘two or less draws to decision threshold’, which was found to be most reliably associated with delusions^34^ and widely used in previous studies^32,46,82^. However, concerns have been raised about the Beads Task as a measurement of JTC cognitive bias since patients’ tendency to ‘over-adjustment’ may be explained by miscomprehension of the test^83^. In keeping with this, we did not consider the Beads Task as a continuous variable due to power-related issues detailed below.

In order to assess mentalizing or *Theory of Mind (ToM)*, two instruments were administered. First, two different stories from the *Hinting Task*^53^ Spanish version^84^, which was found to have acceptable internal consistency (α = 0.64)^84^, were used in each assessment to avoid learning. Scores therefore ranged from 0 to 4. Second, the *Emotions Recognition Test Faces* activity (ERTF)^51,52^, which is composed of 20 different photographs showing people’s facial expressions, evaluated patients’ ability to recognise people’ emotions between two given options. Each right answer is given a score of 1, which can be summed up to create total scores ranging from 0 to 20; higher scores indicated better ToM performance.

Although the Spanish version^85^ of the Positive and Negative Syndrome Scale (PANSS)^86^ which revealed three psychopathological dimensions—positive, negative and disorganised^85^, was used to assess *symptoms severity*, five symptomatic dimensions, namely positive, negative, disorganisation, excitement and depression, were taken based on a more updated review of previous PANSS factor analysis studies^56^ as follows: positive (P1, P3, G5, G9), negative (N1, N2, N3, N4, N6, G7), disorganisation (P2, N5, G11), excitement (P4, P7, G8, G14) and depression (G2, G3, G6). Specifically, Depressive symptoms severity was also measured with the Spanish version^87^ of the Calgary Depression Scale for Schizophrenia (CDSS)^59^, which is a 9-item structured interview enquiring about symptoms of depression, each of which is scored within a 4-point Likert scale ranging from 0 (absent) to 3 (severe). Total CDSS scores therefore range from 0 to 27. Based on the first Kraepelinian classification of endogenous psychoses^88^ schizophrenia has long been considered as a ‘non-affective’ psychotic illness. However, not only recent research has supported the dimensional model of psychoses^89^, but also mania and depression symptoms have been shown to be intrinsic to schizophrenia^70,90,91^.

*Functioning* was recorded through the Global Assessment of Functioning (GAF)^92^ and the 12-item version of the World Health Organization Disability Schedule (WHODAS)^93^, while the Spanish adaptation^94^ of the Satisfaction Life Domains Scale (SLDS)^95^ measured quality of life.

#### Additional variables

We collected baseline data on age, gender, education level, marital status, employment status, living status, ICD-10 diagnosis, previous suicidal behaviour (present/absent), illness duration and number of previous admissions, number of antipsychotics (one or more than one), being on long-acting injections (present/absent), taking clozapine (present/absent) and chlorpromazine equivalents (mg), premorbid adjustment assessed with the Premorbid Adjustment Scale (PAS)^96^ and neurocognition. The Wechsler Adult Intelligence Scale (WAIS)-IV -vocabulary subtest-^75^ estimated participants’ IQ and the Trail Making Test (TMT)^97^ assessed executive function, particularly ‘time to complete Task A (in seconds) minus time to complete Task B’, which provides a brief measure of executive function (set maintenance/shifting), whilst controlling for processing speed^9^. We did not report on medication changes over the trial, which were marginal (data available on request).

### Interventions

In addition to treatment as usual (TAU), which consisted of regular face-to-face appointments with the treating consultant psychiatrist, consultant psychologist and registered mental health nurse as appropriate and taking antipsychotic medication, participants were randomised either to receive MCT or to attend a psychoeducation group. Hence, all participants were meant to receive one weekly 45–60-min group session lasting over 8 weeks.

#### Metacognitive training (MCT)

Metacognitive Training (MCT)^58^ addresses positive symptoms of schizophrenia from an indirect approach which seeks to plant the seeds of doubt regarding cognitive biases leading to delusional thoughts. MCT focuses on different topics (Modules) shown by structured powerpoint presentations: Attributional Style (Module 1), Jumping to Conclusions (Modules 2 and 7), Changing Beliefs (Module 3), Empathy (Modules 4 and 6), Memory (Modules 5), Depression and Self-Esteem (Module 8) and two additional modules, namely Self-Esteem (Module 9) and Stigma (Module 10), although Modules 8, 9 and 10 were delivered together as one session. Although subject to future investigation, MCT was found to be efficacious for those who attended (at least) four sessions^38,43^. As a result, only those who attended four or more sessions were analysed.

#### Psychoeducation control group

Controls attended eight weekly psychoeducation group sessions on: (1) basic and (2) instrumental activities of daily living, (3) physical health, (4) newspapers-based work, (5) emotions, (6) illness, (7) social and family relationships. One additional session allowed participants to raise further concerns.

Both groups were conducted by a higher-trainee clinical psychologist (ASEM), who received direct training from one co-author of the Spanish version of the MCT manual (MLB). Treatment fidelity was looked at by this researcher (MLB) against the MCT manual criteria (http://www.uke.de/mkt), while a significant exposure of controls to MCT elements was ruled out during two random sessions over a month.

### Statistical analysis

First, we explored baseline between-group differences in sociodemographic, premorbid adjustment, clinical, neurocognitive variables and outcome measures, including insight levels (Table 1). Second, after confirmation of the normal distribution of the co-primary outcomes of the RCT by means of the Kolmogorov–Smirnov test, we conducted Student’s *t* tests to examine between-group differences in the SAI-E and BCIS total and subtotal scores changes from T0 to T1 and from T0 to T2 (as the dependent variable) (Table 2). Third, within-group SAI-E and BCIS score changes from T0 to T1 and from T0 to T2 were also investigated (Tables 3 and 4). Effect sizes (Cohen’s *d*) were calculated for between- and within-group comparisons, which were classified as *small* (*d* < *0.2), medium (d* = 0.5) or *large* (*d* > 0.8)^98^. JTC was the only binary outcome measure so McNemar’s test investigated between-assessment changes in each treatment group.

It is true that General Linear Mixed Models are particularly useful in longitudinally analysing between-group differences by modelling fixed and random effects. However, our small sample size and limited power, as detailed below, and the ‘normal’ distribution of the dependent variable, namely, SAI-E and BCIS scores ‘changes’ (i.e., whilst controlling for baseline data) led us to use Student’s *t* test for the analyses, which provided a unique *P* value of significance for between-group comparisons at post treatment and at follow-up^99^.

Analyses were performed for those participants who completed at least 4 treatment sessions regardless of the group^38,43^ using the Statistical Package for Social Science version 25.0 (SPSS, IBM Corp.; Armonk, NY, USA). Power calculations indicated that a total sample size of *N* = 102 subjects (*n* = 51 in each treatment arm) at the end of the trial would be needed to detect a medium effect size (*d* = 0.50, α = 5%, 1-β = 80%) for the primary outcome measure (SAI-E total score). As detailed above, recruitment and assessments had to be stopped due to unforeseen circumstances related to the COVID-19 outbreak in Spain in March 2020, which prevented us from reaching the required sample size. On the other hand, given the final underpowered sample size we did not apply corrections for multiple testing since Type I error was very unlikely.

## Supplementary information

MCT group: Baseline differences between those who attended 4 sessions (n=18) and those who did not (n=21)

Table S2. Psychoeducation group: Baseline differences between those who attended 4 sessions (n=16) and those who did not (n=22)

Table S3. Baseline differences between MCT subjects and controls who attended 4 sessions (n=34)

## Data Availability

Data supporting these results are available upon reasonable request to the corresponding author, provided the dataset access policy is complied with.

## References

[1] Escobedo-Aedo PJ (2022). Investigating the role of insight, decision-making and mentalizing in functional outcome in schizophrenia: a cross-sectional study. Behav. Sci..

[2] Lincoln TM, Lüllmann E, Rief W (2007). Correlates and long-term consequences of poor insight in patients with schizophrenia. A systematic review. Schizophr. Bull..

[3] Carpenter WT, Strauss JS, Bartko JJ (1973). Flexible system for the diagnosis of schizophrenia: report from the WHO International Pilot Study of Schizophrenia. Science.

[4] Amador XF (1994). Awareness of illness in schizophrenia and schizoaffective and mood disorders. Arch Gen. Psychiatry.

[5] Ayesa-Arriola R (2014). Lack of insight 3 years after first-episode psychosis: an unchangeable illness trait determined from first presentation?. Schizophr. Res..

[6] David AS (1990). Insight and psychosis. Br. J. Psychiatry.

[7] David, A. S. Insight and psychosis: the next 30 years. *Br. J. Psychiatry* **217**, 521–523 (2019). 10.1192/bjp.2019.217 31685039

[8] Morgan KD (2010). Insight, grey matter and cognitive function in first-onset psychosis. Br. J. Psychiatry.

[9] Wiffen BDR (2012). Are there specific neuropsychological deficits underlying poor insight in first episode psychosis?. Schizophr. Res..

[10] Henry C, Ghaemi SN (2004). Insight in psychosis: a systematic review of treatment interventions. Psychopathology.

[11] Pijnenborg GHM, van Donkersgoed RJM, David AS, Aleman A (2013). Changes in insight during treatment for psychotic disorders: a meta-analysis. Schizophr. Res..

[12] Lopez-Morinigo J-D (2020). Can metacognitive interventions improve insight in schizophrenia spectrum disorders? A systematic review and meta-analysis. Psychol. Med..

[13] Flavell JH (1979). Metacognition and cognitive monitoring: a new area of cognitive-developmental inquiry. Am. Psychologist.

[14] Wells Adrian (1999). Metacognition and cognitive-behaviour therapy: a special issue. Clin. Psychol. Psychother..

[15] Beck AT, Baruch E, Balter JM, Steer RA, Warman DM (2004). A new instrument for measuring insight: the Beck Cognitive Insight Scale. Schizophr. Res..

[16] Lysaker PH, Pattison ML, Leonhardt BL, Phelps S, Vohs JL (2018). Insight in schizophrenia spectrum disorders: relationship with behavior, mood and perceived quality of life, underlying causes and emerging treatments: world psychiatry. World Psychiatry.

[17] Van Camp LSC, Sabbe BGC, Oldenburg JFE (2017). Cognitive insight: a systematic review. Clin. Psychol. Rev..

[18] van Oosterhout B (2016). Metacognitive training for schizophrenia spectrum patients: a meta-analysis on outcome studies. Psychol. Med..

[19] van Oosterhout B (2014). Metacognitive group training for schizophrenia spectrum patients with delusions: a randomized controlled trial. Psychol. Med..

[20] Eichner C, Berna F (2016). Acceptance and efficacy of metacognitive training (MCT) on positive symptoms and delusions in patients with schizophrenia: a meta-analysis taking into account important moderators. Schizophr. Bull..

[21] Jiang J, Zhang L, Zhu Z, Li W, Li C (2015). Metacognitive training for schizophrenia: a systematic review. Shanghai Arch Psychiatry.

[22] Liu Y-C, Tang C-C, Hung T-T, Tsai P-C, Lin M-F (2018). The efficacy of metacognitive training for delusions in patients with schizophrenia: a meta-analysis of randomized controlled trials informs evidence-based practice. Worldviews on Evidence-Based Nursing.

[23] Moritz S (2014). Sowing the seeds of doubt: a narrative review on metacognitive training in schizophrenia. Clin. Psychol. Rev..

[24] Philipp R (2019). Effectiveness of metacognitive interventions for mental disorders in adults—a systematic review and meta-analysis (METACOG). Clin. Psychol. Psychother..

[25] van Oosterhout B (2016). Letter to the editor: should we focus on quality or quantity in meta-analyses?. Psychol. Med..

[26] Penney D (2022). Immediate and sustained outcomes and moderators associated with metacognitive training for psychosis: a systematic review and meta-analysis. JAMA Psychiatry.

[27] Birulés, I. et al. Cognitive insight in first-episode psychosis: changes during metacognitive training. *J. Pers. Med.* **10**. 10.3390/jpm10040253 (2020). 10.3390/jpm10040253 PMC7711871 33260823

[28] Bora E, Pantelis C (2013). Theory of mind impairments in first-episode psychosis, individuals at ultra-high risk for psychosis and in first-degree relatives of schizophrenia: systematic review and meta-analysis. Schizophr. Res..

[29] Moritz S (2017). A two-stage cognitive theory of the positive symptoms of psychosis. Highlighting the role of lowered decision thresholds. J. Behav. Ther. Exp. Psychiatry.

[30] Brar PS, Sass L, Beck D, Kalarchian MA (2022). Metacognitive training for schizophrenia: a scoping review and phenomenological evaluation. Psychosis.

[31] Lopez-Morinigo J-D (2022). Investigating the contribution of decision-making, cognitive insight, and theory of mind in insight in schizophrenia: a cross-sectional study. Psychopathology.

[32] Falcone MA (2015). Jumping to conclusions, neuropsychological functioning, and delusional beliefs in first episode psychosis. Schizophr Bull.

[33] Garety, P. Reasoning and delusions. *Br. J. Psychiatry Suppl*. **159**, 14–18 (1991). 1840774

[34] Garety PA (2005). Reasoning, emotions, and delusional conviction in psychosis. J. Abnorm. Psychol..

[35] Falcone MA (2015). Jumping to conclusions and the persistence of delusional beliefs in first episode psychosis. Schizophr. Res..

[36] Baron-Cohen S, Leslie AM, Frith U (1985). Does the autistic child have a ‘theory of mind’?. Cognition.

[37] Weng Y, Lin J, Ahorsu DK, Tsang HWH (2022). Neuropathways of theory of mind in schizophrenia: a systematic review and meta-analysis. Neurosci. Biobehav. Rev..

[38] Balzan RP, Mattiske JK, Delfabbro P, Liu D, Galletly C (2019). Individualized metacognitive training (MCT+) reduces delusional symptoms in psychosis: a randomized clinical trial. Schizophr. Bull..

[39] Briki M (2014). Metacognitive training for schizophrenia: a multicentre randomised controlled trial. Schizophr. Res..

[40] Favrod J (2014). Sustained antipsychotic effect of metacognitive training in psychosis: a randomized-controlled study. Eur. Psychiatry.

[41] Gawęda Ł, Krężołek M, Olbryś J, Turska A, Kokoszka A (2015). Decreasing self-reported cognitive biases and increasing clinical insight through meta-cognitive training in patients with chronic schizophrenia. J. Behav. Ther. Exp. Psychiatry.

[42] Kuokkanen R, Lappalainen R, Repo-Tiihonen E, Tiihonen J (2014). Metacognitive group training for forensic and dangerous non-forensic patients with schizophrenia: a randomised controlled feasibility trial. Crim. Behav. Mental Health.

[43] Ahuir M (2018). Improvement in cognitive biases after group psychoeducation and metacognitive training in recent-onset psychosis: a randomized crossover clinical trial. Psychiatry Res..

[44] Favrod J (2014). Sustained antipsychotic effect of metacognitive training in psychosis: a randomized-controlled study. Eur. Psychiatry.

[45] Mehl S, Werner D, Lincoln TM (2015). Does Cognitive Behavior Therapy for psychosis (CBTp) show a sustainable effect on delusions? A meta-analysis. Front. Psychol..

[46] Ochoa S (2017). Randomized control trial to assess the efficacy of metacognitive training compared with a psycho-educational group in people with a recent-onset psychosis. Psychol. Med..

[47] Salas-Sender M (2020). Gender differences in response to metacognitive training in people with first-episode psychosis. J. Consult. Clin. Psychol..

[48] Lüdtke T, Pult LK, Schröder J, Moritz S, Bücker L (2018). A randomized controlled trial on a smartphone self-help application (Be Good to Yourself) to reduce depressive symptoms. Psychiatry Res..

[49] Andreou C (2017). Individualized metacognitive therapy for delusions: a randomized controlled rater-blind study. J. Behav. Ther. Exp. Psychiatry.

[50] Aghotor J, Pfueller U, Moritz S, Weisbrod M, Roesch-Ely D (2010). Metacognitive training for patients with schizophrenia (MCT): feasibility and preliminary evidence for its efficacy. J. Behav. Ther. Exp. Psychiatry.

[51] Baron-Cohen S, Wheelwright S, Jolliffe T (1997). Is there a ‘language of the eyes’? Evidence from normal adults, and adults with autism or Asperger syndrome. Visual Cognition.

[52] Huerta-Ramos E (2021). Translation and validation of Baron Cohen’s face test in a general population from Spain. Actas Esp Psiquiatr.

[53] Corcoran R, Mercer G, Frith CD (1995). Schizophrenia, symptomatology and social inference: Investigating “theory of mind” in people with schizophrenia. Schizophr. Res..

[54] Langdon R, Still M, Connors MH, Ward PB, Catts SV (2014). Theory of mind in early psychosis. Early Interv. Psychiatry.

[55] Sprong M, Schothorst P, Vos E, Hox J, van Engeland H (2007). Theory of mind in schizophrenia: meta-analysis. Br. J. Psychiatry.

[56] Wallwork RS, Fortgang R, Hashimoto R, Weinberger DR, Dickinson D (2012). Searching for a consensus five-factor model of the positive and negative syndrome Scale for schizophrenia. Schizophr. Res..

[57] Sarraf L, Lepage M, Sauvé G (2022). The clinical and psychosocial correlates of self-stigma among people with schizophrenia spectrum disorders across cultures: a systematic review and meta-analysis. Schizophr. Res..

[58] Moritz S, Woodward TS (2007). Metacognitive training for schizophrenia patients (MCT): a pilot study on feasibility, treatment adherence, and subjective efficacy. German J. Psychiatry.

[59] Addington D, Addington J, Maticka-Tyndale E, Joyce J (1992). Reliability and validity of a depression rating scale for schizophrenics. Schizophr. Res..

[60] Belvederi Murri M (2015). Is good insight associated with depression among patients with schizophrenia? Systematic review and meta-analysis. Schizophr. Res..

[61] Mintz AR, Dobson KS, Romney DM (2003). Insight in schizophrenia: a meta-analysis. Schizophr. Res..

[62] Drake RE, Cotton PG (1986). Depression, hopelessness and suicide in chronic schizophrenia. Br. J. Psychiatry.

[63] Ghaemi SN (2007). Feeling and time: the phenomenology of mood disorders, depressive realism, and existential psychotherapy. Schizophr. Bull..

[64] Lysaker PH, Roe D, Yanos PT (2007). Toward understanding the insight paradox: internalized stigma moderates the association between insight and social functioning, hope, and self-esteem among people with schizophrenia spectrum disorders. Schizophr. Bull..

[65] Lysaker PH, Pattison ML, Leonhardt BL, Phelps S, Vohs JL (2018). Insight in schizophrenia spectrum disorders: relationship with behavior, mood and perceived quality of life, underlying causes and emerging treatments. World Psychiatry.

[66] Palmer EC, Gilleen J, David AS (2015). The relationship between cognitive insight and depression in psychosis and schizophrenia: a review and meta-analysis. Schizophr. Res..

[67] García-Mieres H, De, Jesús-Romero R, Ochoa S, Feixas G, IDENTITY group (2020). Beyond the cognitive insight paradox: Self-reflectivity moderates the relationship between depressive symptoms and general psychological distress in psychosis. Schizophr. Res..

[68] Ayesa-Arriola R (2018). The dynamic relationship between insight and suicidal behavior in first episode psychosis patients over 3-year follow-up. Eur. Neuropsychopharmacol..

[69] Lopez-Morinigo J-D (2019). Insight and risk of suicidal behaviour in two first-episode psychosis cohorts: Effects of previous suicide attempts and depression. Schizophr. Res..

[70] Upthegrove R, Marwaha S, Birchwood M (2017). Depression and schizophrenia: cause, consequence, or trans-diagnostic issue?. Schizophr. Bull..

[71] Pijnenborg GHM (2015). Differential effects of antipsychotic drugs on insight in first episode schizophrenia: data from the European First-Episode Schizophrenia Trial (EUFEST). Eur. Neuropsychopharmacol..

[72] Lam KCK (2015). Metacognitive training (MCT) for schizophrenia improves cognitive insight: a randomized controlled trial in a Chinese sample with schizophrenia spectrum disorders. Behav. Res. Ther..

[73] Sheehan DV (1998). The Mini-International Neuropsychiatric Interview (M.I.N.I.): the development and validation of a structured diagnostic psychiatric interview for DSM-IV and ICD-10. J. Clin. Psychiatry.

[74] World Health Organization. *The ICD-10 Classification of Mental and Behavioural Disorders: Diagnostic Criteria for Research* (World Health Organization, 1993).

[75] Wechsler D (1981). The psychometric tradition: developing the wechsler adult intelligence scale. Contemporary Educ. Psychol..

[76] Lopez-Morinigo J-D (2020). Study protocol of a randomised clinical trial testing whether metacognitive training can improve insight and clinical outcomes in schizophrenia. BMC Psychiatry.

[77] Soriano-Barceló J (2016). Insight assessment in psychosis and psychopathological correlates: validation of the Spanish version of the Schedule for Assessment of Insight - Expanded Version. Eur. J. Psychiatry.

[78] Kemp, R. & David, A. S. Insight and compliance. Chronic mental illness. in *Treatment Compliance and the Therapeutic Alliance* (ed. Blackwell, B.) Vol. 5, 61–84 (Gordon and Breach, 1997).

[79] Sanz M, Constable G, Lopez-Ibor I, Kemp R, David AS (1998). A comparative study of insight scales and their relationship to psychopathological and clinical variables. Psychol. Med..

[80] Gutiérrez-Zotes JA (2012). Spanish adaptation of the Beck Cognitive Insight Scale (BCIS) for schizophrenia. Actas Esp. Psiquiatr..

[81] Brett-Jones J, Garety P, Hemsley D (1987). Measuring delusional experiences: a method and its application. Br. J. Clin. Psychol..

[82] O’Connor JA (2017). Can cognitive insight predict symptom remission in a first episode psychosis cohort?. BMC Psychiatry.

[83] Balzan RP, Delfabbro PH, Galletly CA, Woodward TS (2012). Over-adjustment or miscomprehension? A re-examination of the jumping to conclusions bias. Aust N Z J Psychiatry.

[84] Gil D, Fernández-Modamio M, Bengochea R, Arrieta M (2012). Adaptation of the Hinting Task theory of the mind test to Spanish. *Revista de Psiquiatría y*. Salud Mental.

[85] Peralta, V. & Cuesta, M. Validation of positive and negative symptom scale (PANSS) in a sample of Spanish schizophrenic patients. *Psychiatry Res*. **53**, 31–40 (1994). 10.1016/0165-1781(94)90093-0 7991730

[86] Kay SR, Fiszbein A, Opler LA (1987). The positive and negative syndrome scale (PANSS) for Schizophrenia. Schizophr. Bull..

[87] Sarró S (2004). Cross-cultural adaptation and validation of the Spanish version of the Calgary Depression Scale for Schizophrenia. Schizophr. Res..

[88] Jablensky A (2007). Living in a Kraepelinian world: Kraepelin’s impact on modern psychiatry. Hist Psychiatry.

[89] Craddock N, Owen MJ (2010). The Kraepelinian dichotomy—going, going… but still not gone. Br. J. Psychiatry.

[90] Peralta V, Cuesta MJ (2001). How many and which are the psychopathological dimensions in schizophrenia? Issues influencing their ascertainment. Schizophr. Res..

[91] van Os J, Kapur S (2009). Schizophrenia. Lancet.

[92] Endicott J, Spitzer RL, Fleiss JL, Cohen J (1976). The global assessment scale. A procedure for measuring overall severity of psychiatric disturbance. Arch Gen. Psychiatry.

[93] Üstün, T. B. (ed.). *Measuring Health and Disability: Manual for WHO Disability Assessment Schedule WHODAS 2.0* (World Health Organization, 2010).

[94] Carlson J (2009). Adaptation and validation of the quality-of-life scale: satisfaction with life domains scale by Baker and Intagliata. Compr. Psychiatry.

[95] Baker F, Intagliata J (1982). Quality of life in the evaluation of community support systems. Eval Program Plann..

[96] Cannon-Spoor HE, Potkin SG, Wyatt RJ (1982). Measurement of premorbid adjustment in chronic schizophrenia. Schizophr. Bull..

[97] Reitan RM (1958). Validity of the trail making test as an indicator of organic brain damage. Percept. Mot. Skills.

[98] Cohen, J. *Statistical Power Analysis for the Behavioral Sciences*. 2nd edn. (L. Erlbaum Associates, 1988).

[99] Mishra P, Singh U, Pandey CM, Mishra P, Pandey G (2019). Application of student’s t-test, analysis of variance, and covariance. Ann. Card. Anaesth..

