# Supplementary material for: A pilot 1-year follow-up randomised controlled trial comparing metacognitive training to psychoeducation in schizophrenia: effects on insight
Source: Schizophrenia (Heidelb). 2023 Jan 30;9(1):7. doi: 10.1038/s41537-022-00316-x (PMC9886217; doi:10.1038/s41537-022-00316-x)
Supplement: Supplementary file 1 — MCT group: Baseline differences between those who attended 4 sessions (n=18) and those who did not (n=21) [file 41537_2022_316_MOESM1_ESM.doc]

**Table S1. MCT group: Baseline differences between those who attended 4 sessions (n=18) and those who did not (n=21)**

|  | ***Attendees***  ***(n=18)*** | ***non-attendees***  ***(n=21)*** | ***Statistic*** | ***P*** |
| --- | --- | --- | --- | --- |
| *Sociodemographic variables* |  |  |  |  |
| Age (years) | 45.9 ± 10.1 | 45.4 ± 9.6 | *t_37_*=0.17 | .86 |
| Gender (males) | 11 (61.1) | 10 (47.6) | *X^2^_1_*=0.71 | .39 |
| Education level (primary) | 4 (22.2) | 2 (9.5) | *X^2^_1_*=1.20 | .27 |
| Marital status (unmarried) | 16 (88.9) | 19 (90.5) | *X^2^_1_*=0.03 | .87 |
| Employment status (Unemployed) | 13 (72.2) | 15 (71.4) | *X^2^_1_*=0.03 | .96 |
| Living status (alone) | 2 (11.1) | 2 (9.5) | *X^2^_1_*=0.03 | .87 |
| *Premorbid Adjustment (PAS)* |  |  |  |  |
| Childhood | 6.8 ± 4.6 | 6.4 ± 4.7 | *t_37_*=0.30 | .76 |
| Early adolescence | 9.1 ± 5.5 | 8.1 ± 5.2 | *t_37_*=0.55 | .58 |
| Late adolescence | 9.00 ± 5.6 | 8.1 ± 6.0 | *t_33_*=0.44 | .66 |
| *Clinical variables* |  |  |  |  |
| Diagnosis (Schizophrenia) | 10 (55.5) | 13 (61.9) | *X^2^_1_*=0.16 | .69 |
| Duration of illness (>5years) | 15 (83.3) | 18 (85.7) | *X^2^_1_*=0.04 | .84 |
| Previous admissions | 2.6 ± 2.6 | 3.0 ± 3.1 | *t_37_*=-0.42 | .68 |
| Previous suicidal behaviour | 7 (38.9) | 10 (47.6) | *X^2^_1_*=0.30 | .58 |
| *Antipsychotics-related variables* |  |  |  |  |
| Monotherapy | 8 (44.4) | 15 (71.4) | *X^2^_1_*=2.92 | .088 |
| Long-Acting injections | 14 (77.8) | 17 (80.9) | *X^2^_1_*=0.06 | .81 |
| Clozapine | 3 (16.7) | 3 (14.3) | *X^2^_1_*=0.04 | .84 |
| Chlorpromazine equivalents | 502.8 ± 366.5 | 390.5 ± 249.7 | *t_37_*=1.13 | .26 |
| *Neurocognition* |  |  |  |  |
| IQ | 106.7 ± 11.2 | 103.1 ± 12.4 | *t_37_*=0.94 | .35 |
| TMT B-A | 67.4 ± 45.6 | 70.4 ± 36.0 | *t_35_*=-0.23 | .82 |
| ***Co-Primary Outcomes*** |  |  |  |  |
| *Clinical Insight (SAI-E)* |  |  |  |  |
| Illness Recognition | 5.4 ± 2.5 | 5.6 ± 2.7 | *t_37_*=-.0.27 | .78 |
| Symptoms relabelling | 4.8 ± 2.3 | 6.9 ± 2.9 | *t_37_*=-2.44 | .019 |
| Treatment Compliance | 4.5 ± 1.5 | 4.1 ± 1.3 | *t_37_*=0.77 | .45 |
| Total Insight | 14.7 ± 4.9 | 16.6 ± 5.3 | *t_37_*=-1.19 | .24 |
| *Cognitive Insight (BCIS)* |  |  |  |  |
| Self-Reflectiveness | 16.1 ± 5.2 | 16.4 ± 5.8 | *t_36_*=-0.19 | .85 |
| Self-Certainty | 7.2 ± 3.5 | 7.6 ± 3.9 | *t_35_*=-0.29 | .77 |
| Composite Index | 9.1 ± 7.9 | 8.9 ± 7.3 | *t_34_*=0.09 | .93 |
| ***Secondary Outcomes*** |  |  |  |  |
| *Symptomatic severity* |  |  |  |  |
| PANSS-Positive | 7.7 ± 2.7 | 9.3 ± 4.3 | *t_37_*=-1.32 | .19 |
| PANSS-Negative | 12.9 ± 5.9 | 15.5 ± 5.5 | *t_37_*=-1.39 | .17 |
| PANSS-Disorganisation | 5.2 ± 3.2 | 6.4 ± 2.3 | *t_37_*=-1.31 | .19 |
| PANSS-Mania | 6.4 ± 2.1 | 6.6 ± 1.3 | *t_37_*=-0.33 | .74 |
| PANSS-Depression | 7.9 ± 2.9 | 6.8 ± 2.3 | *t_37_*=1.31 | .19 |
| CDSS-Total | 3.5 ± 3.9 | 3.8 ± 3.2 | *t_37_*=-0.23 | .82 |
| *Jumping to Conclusions (JTC)* |  |  |  |  |
| JTC_85:15 | 9 (50.0) | 14 (66.7) | *X^2^_1_*=0.42 | .52 |
| JTC_60:40 | 5 (27.8) | 12 (57.1) | *X^2^_1_*=2.45 | .12 |
| Theory of Mind (ToM) |  |  |  |  |
| Hinting Task | 2.3 ± 1.4 | 2.3 ± 1.0 | *t_37_*=0.12 | .90 |
| ERTF | 16.6 ± 2.2 | 16.4 ± 1.8 | *t_37_*=0.19 | .84 |
| *Functioning* |  |  |  |  |
| GAF | 64.6 ± 9.5 | 60.6 ± 5.7 | *t_37_*=1.59 | .12 |
| WHODAS | 13.9 ± 7.5 | 15.0 ± 11.0 | *t_37_*=-0.36 | .72 |
| SLDS | 81.7 ± 10.8 | 81.2 ± 10.2 | *t_34_*=0.14 | .89 |

MCT: Metacognitive Training. PSE: Psychoeducation. PAS: Premorbid Adjustment Scale (Cannon-Spoor et al., 1982). SAI-E: Schedule for Assessment of Insight, Expanded Version (Kemp & David, n.d.). BCIS: Beck Cognitive Insight Scale (Beck et al., 2004). PANSS: Positive and Negative Syndrome Scale for Schizophrenia (Kay et al., 1987). CDSS: Calgary Depression Scale for Schizophrenia (Addington et al., 1992). ERTF: Emotions Recognition Test Faces (Baron-Cohen et al., 1997). GAF: General Assessment of Functioning (Endicott et al., 1976). WHODAS: World Health Organization Disability Schedule (Üstün, 2010). SLDS: Satisfaction Life Domains Scale (Carlson et al., 2009).
