# Supplementary material for: A pilot 1-year follow-up randomised controlled trial comparing metacognitive training to psychoeducation in schizophrenia: effects on insight
Source: Schizophrenia (Heidelb). 2023 Jan 30;9(1):7. doi: 10.1038/s41537-022-00316-x (PMC9886217; doi:10.1038/s41537-022-00316-x)
Supplement: Supplementary file 2 — Table S2. Psychoeducation group: Baseline differences between those who attended 4 sessions (n=16) and those who did not (n=22) [file 41537_2022_316_MOESM2_ESM.doc]

**Table S2. Psychoeducation group: Baseline differences between those who attended 4 sessions (n=16) and those who did not (n=22)**

|  | ***Attendees***  ***(n=16)*** | ***non-attendees***  ***(n=22)*** | ***Statistic*** | ***P*** |
| --- | --- | --- | --- | --- |
| *Sociodemographic variables* |  |  |  |  |
| Age (years) | 52.0 ± 9.8 | 48.2 ± 8.8 | *t_36_*=1.26 | .22 |
| Gender (males) | 7 (43.7) | 13 (59.1) | *X^2^_1_*=0.87 | .35 |
| Education level (primary) | 2 (12.5) | 5 (22.7) | *X^2^_1_*=0.64 | .42 |
| Marital status (unmarried) | 10 (62.5) | 16 (72.7) | *X^2^_1_*=0.45 | .50 |
| Employment status (Unemployed) | 12 (75.0) | 16 (72.7) | *X^2^_1_*=0.02 | .87 |
| Living status (alone) | 2 (12.5) | 2 (9.1) | *X^2^_1_*=0.11 | .73 |
| *Premorbid Adjustment (PAS)* |  |  |  |  |
| Childhood | 4.3 ± 2.0 | 5.5 ± 2.7 | *t_35_*=-1.44 | .16 |
| Early adolescence | 6.6 ± 3.2 | 6.8 ± 4.0 | *t_35_*=-0.20 | .84 |
| Late adolescence | 7.1 ± 3.5 | 6.7 ± 4.4 | *t_34_*=0.23 | .82 |
| *Clinical variables* |  |  |  |  |
| Diagnosis (Schizophrenia) | 11 (68.7) | 14 (63.6) | *X^2^_1_*=0.11 | .74 |
| Duration of illness (>5years) | 14 (87.5) | 22 (100.0) | *X^2^_1_*=2.90 | .088 |
| Previous admissions | 4.6 ± 6.6 | 3.8 ± 3.1 | *t_35_*=-0.53 | .60 |
| Previous suicidal behaviour | 7 (43.7) | 7 (31.8) | *X^2^_1_*=0.57 | .45 |
| *Antipsychotics-related variables* |  |  |  |  |
| Monotherapy | 7 (43.7) | 15 (68.1) | *X^2^_1_*=2.27 | .13 |
| Long-Acting injections | 10 (62.5) | 11 (50.0) | *X^2^_1_*=0.58 | .44 |
| Clozapine | 3 (18.7) | 3 (13.6) | *X^2^_1_*=0.18 | .67 |
| Chlorpromazine equivalents | 431.2 ± 308.5 | 482.9 ± 441.4 | *t_36_*=-0.40 | .69 |
| *Neurocognition* |  |  |  |  |
| IQ | 109.7 ± 12.0 | 100.7 ± 10.1 | *t_36_*=2.50 | .017 |
| TMT B-A | 57.6 ± 32.3 | 77.6 ± 56.4 | *t_32_*=-1.22 | .23 |
| ***Co-Primary Outcomes*** |  |  |  |  |
| *Clinical Insight (SAI-E)* |  |  |  |  |
| Illness Recognition | 6.0 ± 3.0 | 4.6 ± 2.6 | *t_36_*=1.50 | .14 |
| Symptoms relabelling | 6.0 ± 1.9 | 5.7 ± 3.4 | *t_36_*=0.29 | .77 |
| Treatment Compliance | 4.9 ± 1.5 | 3.9 ± 1.8 | *t_36_*=1.75 | .089 |
| Total Insight | 16.9 ± 4.7 | 14.3 ± 5.9 | *t_36_*=1.45 | .15 |
| *Cognitive Insight (BCIS)* |  |  |  |  |
| Self-Reflectiveness | 15.6 ± 4.7 | 13.6 ± 4.5 | *t_34_*=1.28 | .21 |
| Self-Certainty | 6.7 ± 3.0 | 8.8 ± 3.0 | *t_34_*=-1.98 | .056 |
| Composite Index | 8.9 ± 5.1 | 4.5 ± 5.0 | *t_32_*=2.51 | .017 |
| ***Secondary Outcomes*** |  |  |  |  |
| *Symptomatic severity* |  |  |  |  |
| PANSS-Positive | 9.2 ± 3.9 | 7.7 ± 3.4 | *t_36_*=1.26 | .21 |
| PANSS-Negative | 16.0 ± 5.1 | 15.2 ± 6.6 | *t_36_*=0.39 | .70 |
| PANSS-Disorganisation | 6.2 ± 2.2 | 6.3 ± 2.6 | *t_33_*=-0.03 | .98 |
| PANSS-Mania | 5.7 ± 2.0 | 6.2 ± 2.0 | *t_36_*=-0.82 | .42 |
| PANSS-Depression | 6.8 ± 3.1 | 6.3 ± 2.5 | *t_36_*=0.59 | .56 |
| CDSS-Total | 4.7 ± 4.0 | 2.4 ± 4.2 | *t_36_*=1.72 | .094 |
| *Jumping to Conclusions (JTC)* |  |  |  |  |
| JTC_85:15 | 4 (25.0) | 15 (68.2) | *X^2^_1_*=6.91 | .009 |
| JTC_60:40 | 9 (56.2) | 12 (54.5) | *X^2^_1_*=0.11 | .74 |
| Theory of Mind (ToM) |  |  |  |  |
| Hinting Task | 2.4 ± 1.4 | 2.0 ± 1.5 | *t_36_*=0.69 | .50 |
| ERTF | 17.3 ± 2.3 | 17.2 ± 2.3 | *t_36_*=0.17 | .86 |
| *Functioning* |  |  |  |  |
| GAF | 60.9 ± 7.8 | 61.4 ± 6.7 | *t_36_*=-0.21 | .84 |
| WHODAS | 21.3 ± 12.9 | 12.1 ± 8.7 | *t_35_*=2.61 | .013 |
| SLDS | 72.9 ± 12.1 | 83.6 ± 10.8 | *t_34_*=-2.81 | .008 |

MCT: Metacognitive Training. PSE: Psychoeducation. PAS: Premorbid Adjustment Scale (Cannon-Spoor et al., 1982). SAI-E: Schedule for Assessment of Insight, Expanded Version (Kemp & David, n.d.). BCIS: Beck Cognitive Insight Scale (Beck et al., 2004). PANSS: Positive and Negative Syndrome Scale for Schizophrenia (Kay et al., 1987). CDSS: Calgary Depression Scale for Schizophrenia (Addington et al., 1992). ERTF: Emotions Recognition Test Faces (Baron-Cohen et al., 1997). GAF: General Assessment of Functioning (Endicott et al., 1976). WHODAS: World Health Organization Disability Schedule (Üstün, 2010). SLDS: Satisfaction Life Domains Scale (Carlson et al., 2009).
