# Supplementary material for: A pilot 1-year follow-up randomised controlled trial comparing metacognitive training to psychoeducation in schizophrenia: effects on insight
Source: Schizophrenia (Heidelb). 2023 Jan 30;9(1):7. doi: 10.1038/s41537-022-00316-x (PMC9886217; doi:10.1038/s41537-022-00316-x)
Supplement: Supplementary file 3 — Table S3. Baseline differences between MCT subjects and controls who attended 4 sessions (n=34) [file 41537_2022_316_MOESM3_ESM.doc]

**Table S3. Baseline differences between MCT subjects and controls who attended 4 sessions (n=34)**

|  | ***MCT***  ***(n=18)*** | ***PSE***  ***(n=16)*** | ***Statistic*** | ***P*** |
| --- | --- | --- | --- | --- |
| *Sociodemographic variables* |  |  |  |  |
| Age (years) | 45.9 ± 10.1 | 52.0 ± 9.8 | *t_32_*=-1.77 | .086 |
| Gender (males) | 11 (61.1) | 7 (43.7) | *X^2^_1_*=1.02 | .31 |
| Education level (primary) | 4 (22.2) | 2 (12.5) | *X^2^_1_*=0.55 | .46 |
| Marital status (unmarried) | 16 (88.9) | 10 (62.5) | *X^2^_1_*=3.28 | .070 |
| Employment status (Unemployed) | 13 (72.2) | 12 (75.0) | *X^2^_1_*=0.03 | .85 |
| Living status (alone) | 2 (11.1) | 2 (12.5) | *X^2^_1_*=0.02 | .90 |
| *Premorbid Adjustment (PAS)* |  |  |  |  |
| Childhood | 6.8 ± 4.6 | 4.3 ± 1.9 | *t_32_*=2.03 | .050 |
| Early adolescence | 9.1 ± 5.5 | 9.5 ± 3.2 | *t_32_*=1.58 | .12 |
| Late adolescence | 9.00 ± 5.2 | 7.1 ± 3.5 | *t_29_*=1.21 | .11 |
| *Clinical variables* |  |  |  |  |
| Diagnosis (Schizophrenia) | 10 (55.5) | 11 (68.7) | *X^2^_1_*=0.62 | .43 |
| Duration of illness (>5years) | 15 (83.3) | 14 (87.5) | *X^2^_1_*=0.12 | .73 |
| Previous admissions | 2.6 ± 2.6 | 4.6 ± 6.6 | *t_32_*=-1.20 | .24 |
| Previous suicidal behaviour | 7 (38.9) | 7 (43.7) | *X^2^_1_*=0.08 | .77 |
| *Antipsychotics-related variables* |  |  |  |  |
| Monotherapy | 8 (50.0) | 7 (43.7) | *X^2^_1_*=0.01 | .97 |
| Long-Acting injections | 14 (77.8) | 10 (62.5) | *X^2^_1_*=0.95 | .33 |
| Clozapine | 3 (16.7) | 3 (18.7) | *X^2^_1_*=0.02 | .87 |
| Chlorpromazine equivalents | 502.8 ± 366.5 | 431.2 ± 308.6 | *t_32_*=0.61 | .54 |
| *Neurocognition* |  |  |  |  |
| IQ | 106.7 ± 11.2 | 109.7 ± 12.0 | *t_32_*=0.75 | .45 |
| TMT B-A | 67.4 ± 45.6 | 57.6 ± 32.3 | *t_30_*=0.69 | .49 |
| ***Co-Primary Outcomes*** |  |  |  |  |
| *Clinical Insight (SAI-E)* |  |  |  |  |
| Illness Recognition | 5.4 ± 2.5 | 6.0 ± 2.9 | *t_32_*=-.0.64 | .52 |
| Symptoms relabelling | 4.8 ± 2.3 | 6.0 ± 1.9 | *t_32_*=-1.68 | .10 |
| Treatment Compliance | 4.5 ± 1.5 | 4.9 ± 1.5 | *t_32_*=-0.71 | .48 |
| Total Insight | 14.7 ± 4.8 | 16.9 ± 4.7 | *t_32_*=-1.34 | .19 |
| *Cognitive Insight (BCIS)* |  |  |  |  |
| Self-Reflectiveness | 16.1 ± 5.2 | 15.6 ± 4.7 | *t_32_*=0.28 | .78 |
| Self-Certainty | 7.2 ± 3.5 | 6.7 ± 3.0 | *t_30_*=0.43 | .67 |
| Composite Index | 9.1 ± 7.9 | 8.9 ± 5.1 | *t_30_*=0.09 | .92 |
| ***Secondary Outcomes*** |  |  |  |  |
| *Symptomatic severity* |  |  |  |  |
| PANSS-Positive | 7.7 ± 2.7 | 9.2 ± 3.9 | *t_32_*=-1.29 | .20 |
| PANSS-Negative | 12.9 ± 5.9 | 16.0 ± 5.1 | *t_32_*=-1.61 | .12 |
| PANSS-Disorganisation | 5.2 ± 3.2 | 6.2 ± 2.2 | *t_32_*=-1.08 | .29 |
| PANSS-Mania | 6.4 ± 2.1 | 5.7 ± 1.9 | *t_32_*=-0.99 | .33 |
| PANSS-Depression | 7.9 ± 2.9 | 6.8 ± 3.1 | *t_32_*=1.10 | .28 |
| CDSS-Total | 3.5 ± 3.9 | 4.7 ± 4.0 | *t_32_*=-0.88 | .39 |
| *Jumping to Conclusions (JTC)* |  |  |  |  |
| JTC_85:15 | 9 (50.0) | 4 (25.0) | *X^2^_1_*=3.24 | .072 |
| JTC_60:40 | 5 (27.8) | 9 (56.2) | *X^2^_1_*=2.58 | .11 |
| Theory of Mind (ToM) |  |  |  |  |
| Hinting Task | 2.3 ± 1.4 | 2.4 ± 1.4 | *t_32_*=-0.97 | .33 |
| ERTF | 16.6 ± 2.2 | 17.3 ± 2.3 | *t_32_*=-0.08 | .93 |
| *Functioning* |  |  |  |  |
| GAF | 64.6 ± 9.5 | 60.9 ± 7.8 | *t_32_*=1.22 | .23 |
| WHODAS | 13.9 ± 7.5 | 21.3 ± 12.9 | *t_31_*=-2.01 | .047 |
| SLDS | 81.7 ± 10.8 | 72.9 ± 12.1 | *t_31_*=2.22 | .034 |

MCT: Metacognitive Training. PSE: Psychoeducation. PAS: Premorbid Adjustment Scale (Cannon-Spoor et al., 1982). SAI-E: Schedule for Assessment of Insight, Expanded Version (Kemp & David, n.d.). BCIS: Beck Cognitive Insight Scale (Beck et al., 2004). PANSS: Positive and Negative Syndrome Scale for Schizophrenia (Kay et al., 1987). CDSS: Calgary Depression Scale for Schizophrenia (Addington et al., 1992). ERTF: Emotions Recognition Test Faces (Baron-Cohen et al., 1997). GAF: General Assessment of Functioning (Endicott et al., 1976). WHODAS: World Health Organization Disability Schedule (Üstün, 2010). SLDS: Satisfaction Life Domains Scale (Carlson et al., 2009).
